# Synergistic 3D Porous Architectures and Halogen Redox Chemistry for High‐Energy and High‐Power Microbatteries

**DOI:** 10.1002/advs.75956

**Published:** 2026-06-02

**Authors:** Yijia Zhu, Monojit Mondal, Xiaopeng Liu, Nibagani Naresh, Firoz Alam, Mingqing Wang, Buddha Deka Boruah

**Affiliations:** ^1^ Institute For Materials Discovery University College London London UK; ^2^ Department of Electronic and Electrical Engineering University College London London UK

**Keywords:** 3D microbatteries, On‐chip energy storage, Porous nickel scaffolds, Zn‐iodine chemistry, Zn‐ion batteries

## Abstract

Planar on‐chip microbatteries (MBs) with high‐capacity electrodes and environmentally benign architectures are essential for powering next‐generation system‐on‐chip and miniaturized electronic devices. However, their limited areal capacity and poor rate performance remain major barriers to practical deployment. Here, we present a dual strategy that combines 3D porous Ni scaffolds with halogen redox chemistry to overcome these challenges. The 3D Ni scaffolds, fabricated using a dynamic hydrogen bubble template method, enable efficient loading of Zn anodes and polyaniline (PANI) cathodes, yielding more than a 100% enhancement in areal capacity, together with substantial improvements in rate capability and cycling stability in Zn‐ion MBs. Beyond structural engineering, the incorporation of ZnI_2_ into a Zn(CF_3_SO_3_)_2_ gel electrolyte activates reversible halogen redox chemistry (I^−^/I_3_^−^), further elevating performance. Notably, 3D Zn//I_2_ MBs achieve areal capacities of 150 µAh cm^−^^2^, an areal energy of 142.53 µWh cm^−^^2^, and an areal power of 3443.59 µW cm^−^^2^ at high areal currents (∼ 5 mA/cm^2^)—representing a step‐change in performance that outperforms many state‐of‐the‐art on‐chip energy storage systems. Systematic multi‐modal experimental validation reveals that the synergy between 3D electrode structuring and halogen redox chemistry governs ion diffusion, charge‐transfer kinetics, and long‐term durability.

## Graphical abstract

3D porous Ni scaffolds with PANI cathode, Zn anode, and halogen redox chemistry synergistically enhance on‐chip microbattery performance by improving active material loading, Zn^2^^+^ diffusion, and charge‐transfer kinetics. The resulting 3D Zn//I_2_ microbatteries deliver high areal capacity, high energy and power density, and excellent cycling stability, outperforming many state‐of‐the‐art microscale energy storage systems.

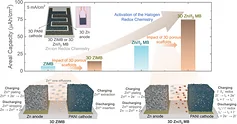

## 1 Introduction

The rapid growth of the Internet of Things (IoT) has placed miniaturization and seamless integration at the core of modern microelectronics. Emerging platforms such as microrobots and microsensors are advancing rapidly and are expected to become an integral part of daily life. Despite volumes of only a few cubic millimeters, these devices are capable of on‐board data processing and wireless communication, making them highly attractive for applications in health monitoring, medical diagnostics, and targeted therapies [1]. For such systems to function effectively, compact, efficient, and reliable power sources are indispensable. As wearable and implantable electronics continue to shrink in size while gaining additional functionalities, the demand for high‐performance micro‐power sources is intensifying [2, 3]. Conventional stacked electrode‐separator microbatteries (MBs) face intrinsic limitations in meeting the stringent integration and power requirements of next‐generation devices. To overcome these challenges, planar MBs with in‐plane patterned electrodes have emerged as a promising alternative [4, 5, 6]. Their unique architecture facilitates monolithic system‐on‐chip integration, enables separator‐free operation, and is fully compatible with standard two‐dimensional (2D) microfabrication processes. These attributes position planar MBs as strong candidates for powering the next generation of miniaturized smart electronics. However, the small active area inherent to planar devices significantly restricts the areal capacity of the micro‐battery, limiting its suitability for applications such as point‐of‐care (PoC) diagnostic platforms, where both high energy density and operational safety are essential requirements. Lithium‐ion batteries (LiBs) currently dominate the energy storage landscape due to their high energy density, elevated operating voltage, and robust cycling stability [7]. Despite these advantages, LiBs face serious challenges in micro‐scale applications: they present well‐known safety risks, rely on costly raw materials, and require sophisticated device assembly in controlled argon environments or dry rooms, which further increases production costs. These limitations highlight the urgent need for alternative battery chemistries that are not only safer but also more cost‐effective. Zinc‐based batteries offer a compelling solution. Benefiting from earth‐abundant raw materials and the ability to operate in aqueous electrolytes, Zn‐based systems provide inherently safer and more environmentally benign chemistry [8, 9, 10]. In addition, Zn anodes deliver a high theoretical capacity of 820 mAh g^−^^1^ (∼ 5855 mAh cm^−^^3^), and their low redox potential (– 0.76 V vs SHE) enables stable operation in aqueous electrolytes [11]. These features collectively make Zn‐based MBs highly promising candidates for next‐generation on‐chip energy storage systems.

Unlike conventional coin cells that employ bulk zinc (Zn) foil anodes and full‐sheet cathode current collectors, MBs are inherently limited by their low mass loading and small device footprint. These constraints make efficient and stable cycling critical to ensuring both performance and long‐term durability [12]. One promising strategy to overcome these challenges is the construction of 3D porous electrode architectures. Such designs provide higher mass loading of cathode materials and increased surface area, thereby improving electrochemical kinetics without enlarging the device footprint. For instance, Liu et al. [13]. demonstrated a 3D nanocone‐array engineered aqueous Zn–Mn MB that achieved a significant areal capacity increase from 30.5 to 51 µAh cm^−^^2^. In our previous work, we have also explored porous Au interdigitated electrodes (IDEs) for Zn‐ion MBs (ZIMBs) [14] and Zn‐ion capacitors [15], achieving substantial improvements in areal capacities. These studies highlight that electrode geometry plays a pivotal role in determining MB performance. More intriguingly, however, beyond geometric engineering, it is possible to significantly alter battery chemistry itself without changing the electrode materials or current collectors—simply through subtle modifications of the electrolyte composition. If such modifications can simultaneously enhance capacity and rate capability, they could dramatically broaden the applicability of Zn‐based MBs, paving the way for their integration into next‐generation miniaturized electronic systems.

In this study, we present a 3D porous Ni scaffold architecture for Zn‐based MBs, designed to overcome the intrinsic limitations of conventional planar devices in terms of capacity, rate capability, and cycling stability. The 3D scaffolds enable efficient active material loading and enhanced electrochemical kinetics, resulting in substantial improvements in areal capacity across both low and high current densities. Compared to conventional Au‐based IDEs, 3D ZIMBs with Ni‐scaffold‐supported PANI cathodes and Zn anodes exhibit over 100% enhancement in areal capacity, together with significant gains in rate performance and long‐term stability. Beyond geometric optimization, additional improvements are achieved through electrolyte engineering by introducing ZnI_2_ into a Zn(CF_3_SO_3_)_2_ gel electrolyte, thereby activating reversible halogen redox chemistry (I^−^/I_3_^−^). This dual strategy yields an areal energy of 142.53 µWh cm^−^^2^ and a peak areal power of 5.68 mW cm^−^^2^, representing a step‐change in performance within the same device footprint. Collectively, these findings establish the integration of 3D Ni scaffolds with halogen redox chemistry as a powerful and practical route toward safe, durable, and high‐performance micropower sources for next‐generation wearable, implantable, and on‐chip electronic systems.

## 2 Results and Discussion

**FIGURE 1:**
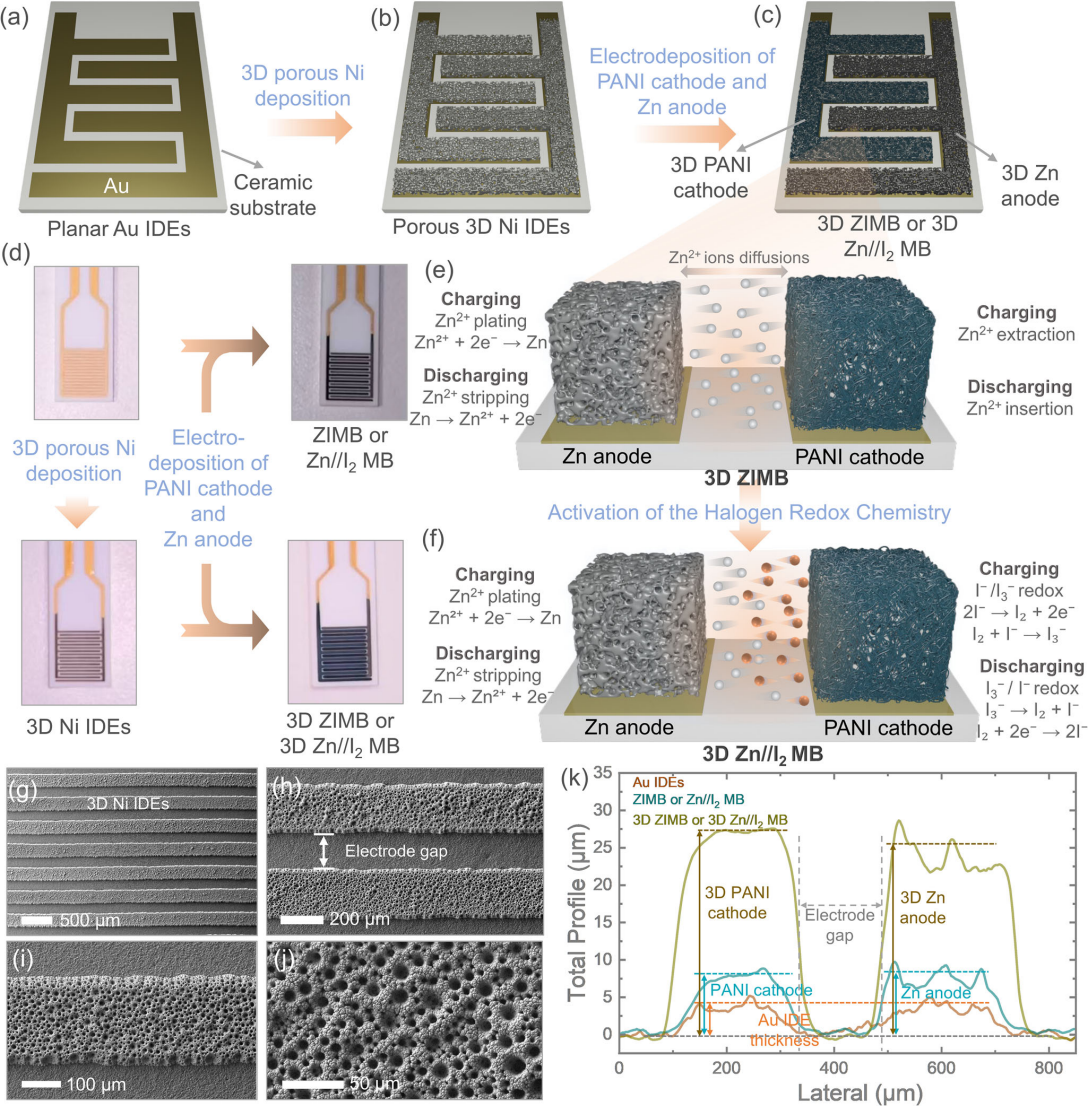
(a–c) Schematic illustration of the fabrication of 3D ZIMBs and 3D Zn//I_2_ MBs, where 3D porous Ni IDEs are formed on Au IDEs using the DHBT, followed by sequential electrodeposition of Zn and PANI active materials. (d) Digital images of the devices at different fabrication stages. (e) Charge storage mechanism of the PANI cathode and Zn anode in ZIMB chemistry with 3 M Zn(CF_3_SO_3_)_2_ gel electrolyte: during charging, Zn^2^^+^ plates onto the Zn anode while Zn^2^^+^ is extracted from the PANI cathode, and the reverse occurs during discharge. (f) Activation of halogen redox chemistry by introducing ZnI_2_ into the 3 M Zn(CF_3_SO_3_)_2_ gel electrolyte without altering electrode compositions: during charging, Zn^2^^+^ plates onto the Zn anode while I^−^/I_3_^−^ redox occurs at the PANI cathode, boosting capacity, rate capability, and overall energy storage within the same device footprint. (g–j) SEM images of the 3D porous Ni IDEs at different magnifications, confirming the porous matrix structure formed via DHBT. (k) Profilometer depth profiles of IDEs, showing the Au IDE thickness (∼ 4.5 µm), Au IDEs with PANI and Zn (∼ 7.5 µm), and the significantly increased thickness of 27 µm when PANI and Zn are deposited onto 3D porous Ni IDEs (∼22 µm).

The fabrication of Zn‐based MBs with 3D Ni scaffolds is schematically illustrated in Figure 1a–c. Unlike conventional Au IDE‐based devices, the incorporation of a 3D porous Ni scaffold, deposited via the dynamic hydrogen bubble template (DHBT) method, provides both structural and functional advantages. This strategy eliminates the need for external templates by using hydrogen bubble evolution to generate a porous metallic network, thereby simplifying the preparation process and offering scalability for large‐area fabrication. As shown in Figure 1d, digital images capture the devices at various fabrication stages. Both PANI cathodes and Zn anodes were deposited on Au IDEs to assemble ZIMBs (based on Zn‐ion chemistry) and Zn//I_2_ MBs (with activated halogen redox chemistry). Equivalent electrode compositions and deposition times were then applied to 3D Ni IDEs to fabricate 3D ZIMBs (Figure 1e) and 3D Zn//I_2_ MBs (Figure 1f). The 3D scaffold is expected to provide improved electron transport pathways and enhanced Zn^2^^+^ diffusion, thereby boosting charge storage, increasing areal capacity, and improving rate performance compared with planar controls. Beyond electrode architecture, electrolyte engineering was employed by introducing ZnI_2_ into a 3 M Zn(CF_3_SO_3_)_2_ gel electrolyte (Figure 1f). This modification activates a halogen redox process (I^−^/I_3_^−^) at the PANI cathode, operating in parallel with the conventional Zn^2^^+^ plating/stripping process at the anode. The combined effect of 3D geometry and halogen redox chemistry yielded remarkable improvements in both energy and power density, without altering the electrode composition or footprint. SEM analysis (Figure 1g–j) confirmed the successful formation of uniformly distributed porous Ni scaffolds on Au IDEs via DHBT. These scaffolds significantly improved electrode performance by enabling higher mass loading of both PANI cathodes and Zn anodes. Even under identical deposition times, Ni‐supported electrodes showed higher electrodeposition current densities, indicating greater active surface area and material accommodation (Figure S1). Consequently, the increased cathode mass loading translated directly into higher areal capacities, as confirmed by electrochemical testing. Profilometry further characterized electrode thickness (Figure 1k). The Au IDE current collector exhibited a thickness of ∼ 4.5 µm, while electrodes loaded with PANI and Zn on Au IDEs reached ∼7.5 µm (including the Au IDE thickness). By comparison, the integration of 3D Ni scaffolds significantly increased the thickness to ∼ 27 µm, with the porous Ni layer contributing ∼ 22 µm (Figure S2). This confirms the effectiveness of the scaffold in providing additional material accommodation for active electrodes.

**FIGURE 2:**
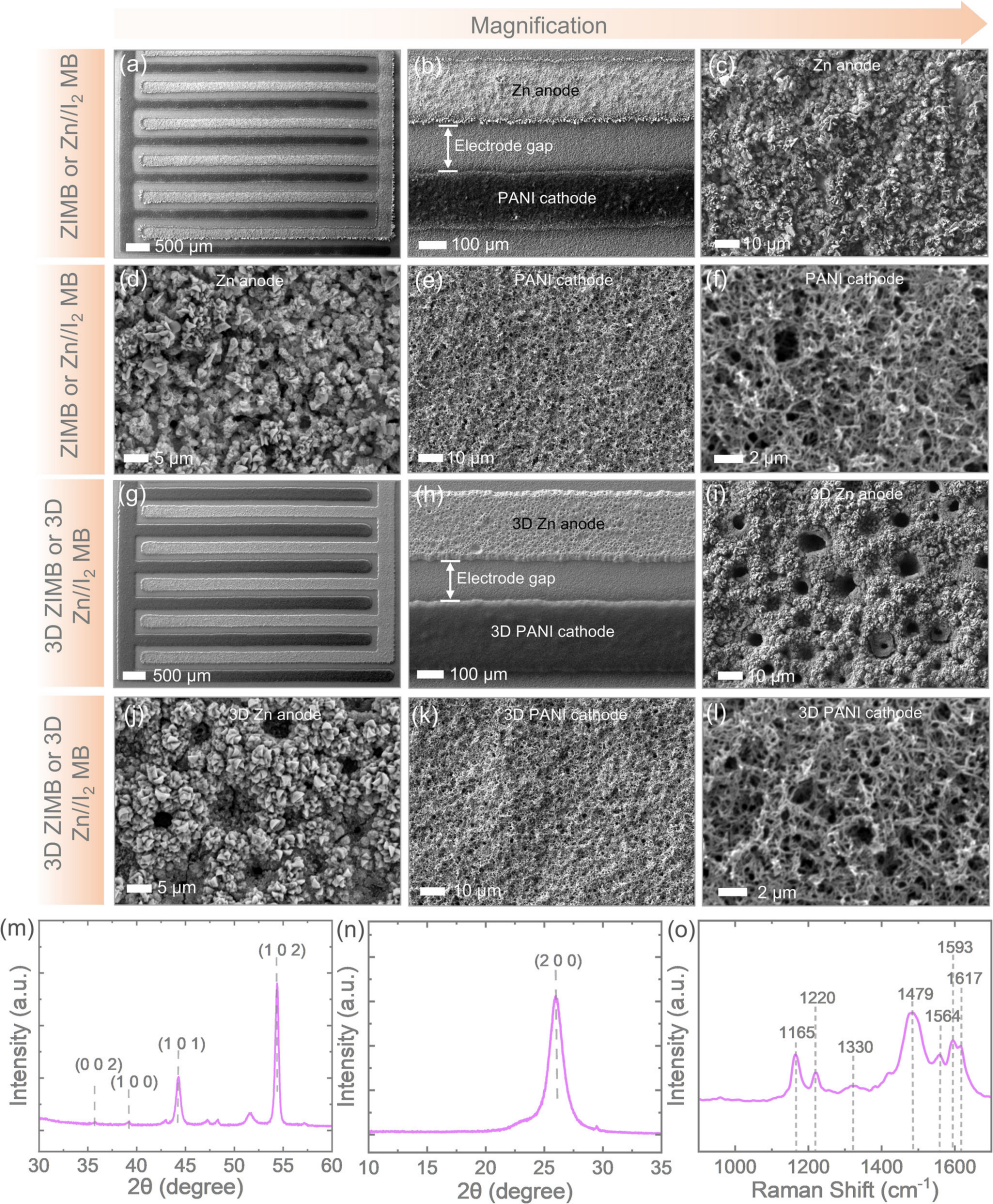
SEM images of (a–f) ZIMB or Zn//I_2_ MB and (g–l) 3D ZIMB or 3D Zn//I_2_ MB at different magnifications. (m, n) XRD pattern of the electrodeposited Zn and PANI. (o) Raman spectrum of electrodeposited PANI.

Figure 2 compares the morphology and structural characterization of ZIMBs (or Zn//I_2_ MBs) and 3D ZIMBs (or 3D Zn//I_2_ MBs). SEM images of the planar ZIMB or Zn//I_2_ MB (Figure 2a–f) show the electrode layout and morphology of the electrodeposited Zn anode and PANI cathode. At low magnification (Figure 2a,b), a clear electrode gap is visible between the Zn and PANI electrodes, confirming the well‐defined interdigitated architecture. Higher magnification images (Figure 2c–f) reveal the granular morphology of the Zn anode and the porous nanowire‐like texture of the PANI cathode. In contrast, the 3D ZIMB or 3D Zn//I_2_ MB (Figure 2g–l) shows similar PANI morphology, but the incorporation of a porous Ni scaffold results in a thicker, more textured Zn anode and a denser porous nanowire PANI morphology cathode. SEM images at different magnifications (Figure 2h–l) highlight the interconnected porous network within both Zn and PANI electrodes. Figure S3 presents cross‐sectional SEM images of Zn and PANI deposited on the 3D porous Ni scaffold. This architecture is expected to facilitate electrolyte penetration, shorten Zn^2^^+^ diffusion pathways, and improve electron transport. Furthermore, the porous scaffold enables higher active material loading, which directly contributes to the enhanced areal capacity of the 3D devices compared to their planar counterparts. Although directly measuring the mass loading of active materials in microelectrodes is challenging, a fair comparison was ensured by fixing the deposition conditions (time and parameters) for Zn and PANI across both planar Au IDEs and 3D Ni‐scaffold‐modified IDEs, with performance evaluated based on identical active device areas, including the electrode gaps. Figure 2m,n present the XRD patterns of the electrode materials, while Figure S4 shows the pattern of the Ni scaffold, confirming the successful synthesis of the target materials. For XRD analysis, Ni and PANI were deposited onto titanium (Ti) substrates, while Zn was deposited onto graphene paper. The Ni scaffold exhibits diffraction peaks at 44.2° and 51.5°, corresponding to the (111) and (200) planes, while additional peaks at 37.8°, 39.7°, and 52.6° arise from the Ti substrate. Zn deposition shows characteristic peaks at 35.7°, 39.2°, 44.3°, and 54.4°, indexed to the (002), (100), (101), and (102) planes of metallic Zn, alongside substrate‐related peaks from graphene in the 47°‐52° range [16]. For PANI, a single broad peak at 26.0° corresponds to the (200) plane, consistent with its semi‐crystalline nature [17]. The Raman spectrum of PANI (Figure 2o) further confirms its successful deposition and electroactive structure. The peak at 1165 cm^−^^1^ corresponds to C–H bending vibrations, while the band at 1220 cm^−^^1^ represents C–N stretching. A strong peak at 1330 cm^−^^1^ is attributed to C–N^+^ stretching, confirming protonated sites. The peaks at 1564 and 1593 cm^−^^1^ correspond to C_α_ = C_β_ in‐plane stretching vibration of carbon atoms in sp^2^ hybridisation and C–C stretching modes of quinoid and benzene units, respectively, while the band at 1617 cm^−^^1^ is associated with C═C/C–C stretching in conjugated rings. Importantly, the peak at 1479 cm^−^^1^ corresponds to C═N stretching in quinoid units, which play a critical role in π‐conjugation and charge delocalization [18, 19]. Together, the XRD and Raman results validate the successful electrodeposition and electrochemical activity of the Zn and PANI electrodes.

**FIGURE 3:**
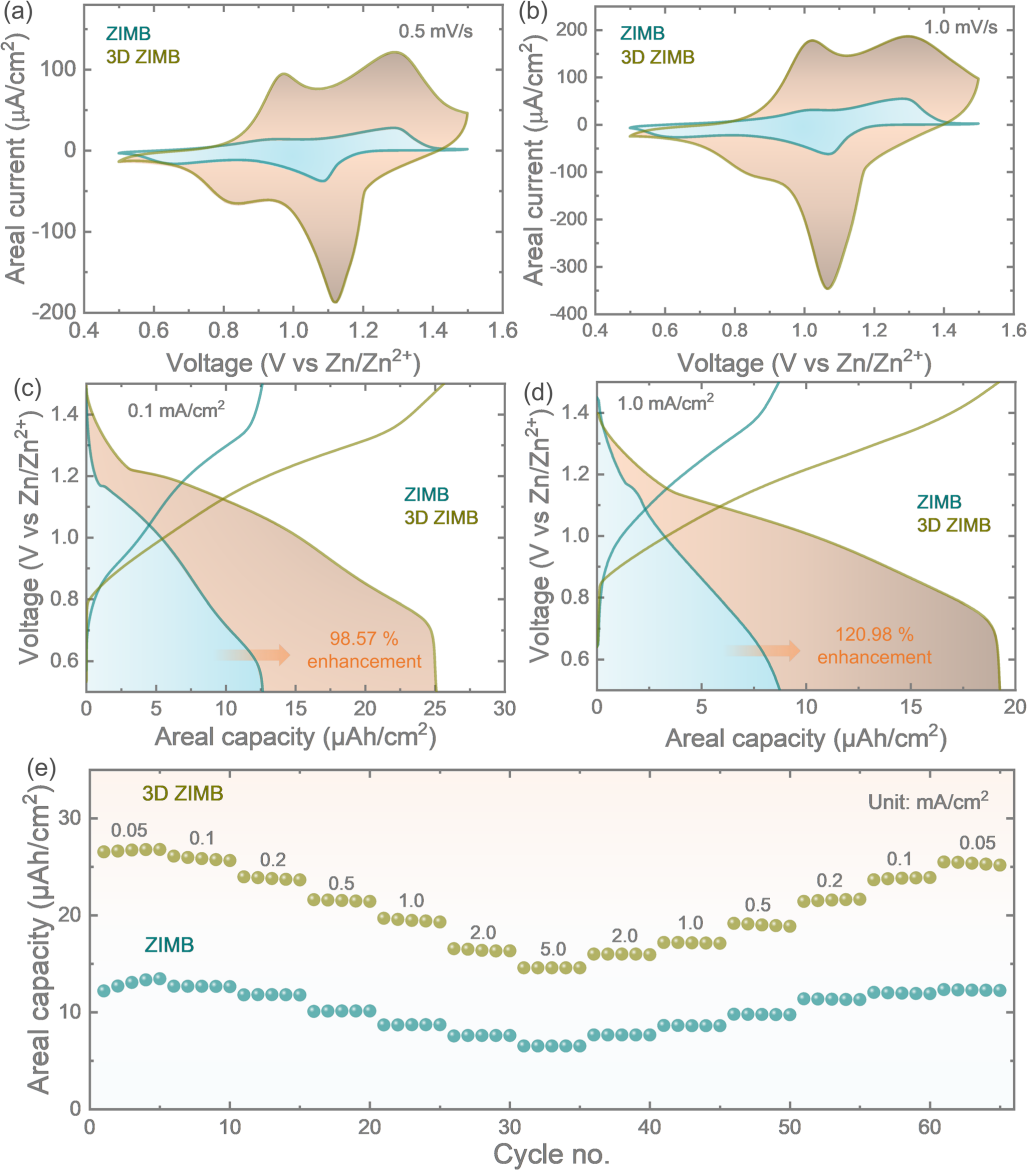
Comparative CV curves of ZIMB and 3D ZIMB at scan rates of (a) 0.5 mV s^−^^1^ and (b) 1.0 mV s^−^^1^, showing significantly higher peak currents with the 3D Ni scaffolds, indicating enhanced charge storage performance despite identical Zn anodes and PANI cathodes. Comparative GCD profiles at (c) 0.1 mA cm^−^^2^ and (d) 1.0 mA cm^−^^2^ reveal 98.57% and 120.98% improvements in areal capacity for 3D ZIMBs, highlighting the role of porous electrodes in improving both storage capacity and rate capability. (e) Rate performance tests further confirm the improved charge storage behavior of 3D ZIMBs compared to planar ZIMBs across all areal currents.

The electrochemical performance of ZIMBs and 3D ZIMBs was first evaluated using a 3 M Zn(CF_3_SO_3_)_2_ PVA gel electrolyte. Figure 3a,b present the comparative cyclic voltammetry (CV) curves of both systems at scan rates of 0.5 and 1.0 mV s^−^^1^. Distinct pairs of redox peaks are observed, corresponding to Zn^2^^+^ ion interactions during the charge storage process. The mechanism involves conjugated C = N bonds, which enable anion binding with oxidized PANI (C–N^+^), while reduced PANI (C–N^−^) accommodates cations. In this case, the triflate anion (CF_3_SO_3_^−^) interacts with oxidized PANI during charging, whereas Zn^2^^+^ ions bind to reduced PANI during discharge [20, 21]. The introduction of 3D Ni scaffolds significantly reduces the overpotential of both major and minor redox peaks, indicating improved charge‐transfer kinetics. Overpotential (Δ*E*  = *E_actual_*  − *E_eq_*) reflects the deviation of the measured electrode potential from the thermodynamic equilibrium potential, where *E_eq_* can be approximated as the midpoint between anodic (*E_pa_*) and cathodic (*E_pc_*) peak potentials: $E_{eq}\approx \frac{E_{pa}+E_{pc}}{2}$ [22, 23]. At a scan rate of 0.5 mV s^−^^1^, the planar ZIMB exhibits major and minor overpotentials of approximately 0.10 V and 0.13 V, respectively. In contrast, the 3D ZIMB shows significantly reduced overpotentials of ∼ 0.08 and ∼ 0.07 V, reflecting improved reaction kinetics. This reduction arises from interfacial optimization provided by the 3D porous architecture, which: increases the effective electrochemically active surface area, shortens ion diffusion pathways and improves Zn^2^^+^ ion diffusion within the electrode (see further), reduces charge‐transfer resistance (see further) at the electrode‐electrolyte interface. As a result, the redox reactions proceed with lower polarization, leading to the observed voltage shifts while preserving the same fundamental redox couples. Supporting Information (Figure S5) further provides CV profiles at scan rates ranging from 0.2 to 1.0 mV s^−^^1^. The integrated CV area, which correlates with storage capacity, is consistently larger for the 3D ZIMBs, demonstrating that the porous 3D structure enables higher charge storage and improved overall capacity relative to planar counterparts. To more intuitively demonstrate the impact of the 3D structure on electrochemical performance, asymmetric 3D Ni scaffolds were introduced, where PANI was deposited on planar Au IDEs, and Zn was deposited onto porous 3D Ni scaffolds to assemble PANI//Zn(3D) MBs as a reference. Additional CV curves of the PANI//Zn(3D) MBs at scan rates ranging from 0.2 to 1.0 mV s^−^^1^ are provided in the Supporting Information (Figure S5b). The CV profiles of these devices retain similar shapes with minimal peak shifts as the scan rate increases. To better understand the charge storage kinetics, we analysed the relative contributions of diffusion‐controlled and capacitive‐controlled processes. Capacitive charge storage, characterized by rapid reversibility, is especially beneficial for achieving high‐rate battery performance. The relationship between peak current (*i_p_*) and scan rate (*v*) in cyclic voltammetry follows the power law: *i_p_* =  *av^b^*, where *a* and *b* are adjustable parameters. A *b*‐value of 0.5 indicates a diffusion‐dominated process, while a value of 1 suggests a capacitive‐controlled mechanism. As shown in Figure S6, the calculated *b*‐values for the anodic peaks of ZIMB, PANI//Zn(3D) MB, and 3D ZIMB are 0.97, 0.97 and 0.74, respectively, while the corresponding cathodic peak *b* values are 0.78, 0.77 and 0.83. The b‐values for the minor peaks follow a similar trend, further confirming the enhanced capacitive behavior.

Galvanostatic charge‐discharge (GCD) measurements were carried out on the MBs across a wide range of areal currents, from 50 µA cm^−^^2^ to 5 mA cm^−^^2^, within a voltage window of 0.5 – 1.5 V (Figure S7). Consistent with the comparative CV results in Figure 3a,b, the 3D ZIMBs exhibited significantly higher areal capacities than planar ZIMBs at all areal currents. At 0.1 mA cm^−^^2^ (Figure 3c), the capacity of the 3D ZIMB was enhanced by 98.57% compared with the planar ZIMB, while at 1.0 mA cm^−^^2^ (Figure 3d), the improvement increased to 120.98%. These results confirm that introducing 3D porous Ni scaffolds not only enhances capacity but also markedly improves rate capability. For comparison, the asymmetric PANI//Zn(3D) MB delivered an areal capacity of 12.70 µAh cm^−^^2^ at 0.1 mA cm^−^^2^ (Figure S7d). This observation highlights that the 3D scaffold is particularly critical on the PANI cathode side. Since Zn possesses a high theoretical capacity (820 mAh g^−^^1^), depositing Zn onto either planar Au IDEs or 3D porous Ni scaffolds does not lead to significant changes in overall device capacity. Instead, the areal capacities of Zn‐based MBs are strongly dictated by the cathode, underscoring the importance of effective PANI loading for maximizing charge storage within the restricted footprint of MBs. What's more, the influence of the thickness of Ni scaffold is also studied. As shown in Figure S8, the areal capacities of the 3D ZIMB with a 20 s Ni scaffold on the cathode side are 23.9, 23.16, 21.57, 18.28, 16.89, 15.75, and 14.06 µAh cm^−^^2^ at current densities of 0.05, 0.1, 0.2, 0.5, 1.0, 2.0, and 5.0 mA cm^−^^2^, respectively. These values are slightly lower than those obtained with the 40 s Ni scaffold, but remain significantly higher than those of conventional planar PANI//Zn MBs. This indicates that increasing the scaffold thickness can help accommodate more active material and improve the overall areal capacity. However, when the thickness was further increased to 60 s deposition, the open‐circuit voltage (OCV) of the MBs dropped below 0.5 V, which is close to the lower voltage limit for charge‐discharge cycling and thus limits stable cycling electrochemical operation. As shown in Figure 3e, the 3D ZIMBs also demonstrate superior rate performance. After high‐rate cycling, capacity retention upon returning to 0.05 mA cm^−^^2^ was 91.01% for the planar ZIMB, compared with 96.04% for the 3D ZIMB. These results further highlight the role of 3D porous scaffolds in enhancing both the durability and rate capability of 3D ZIMBs.

**FIGURE 4:**
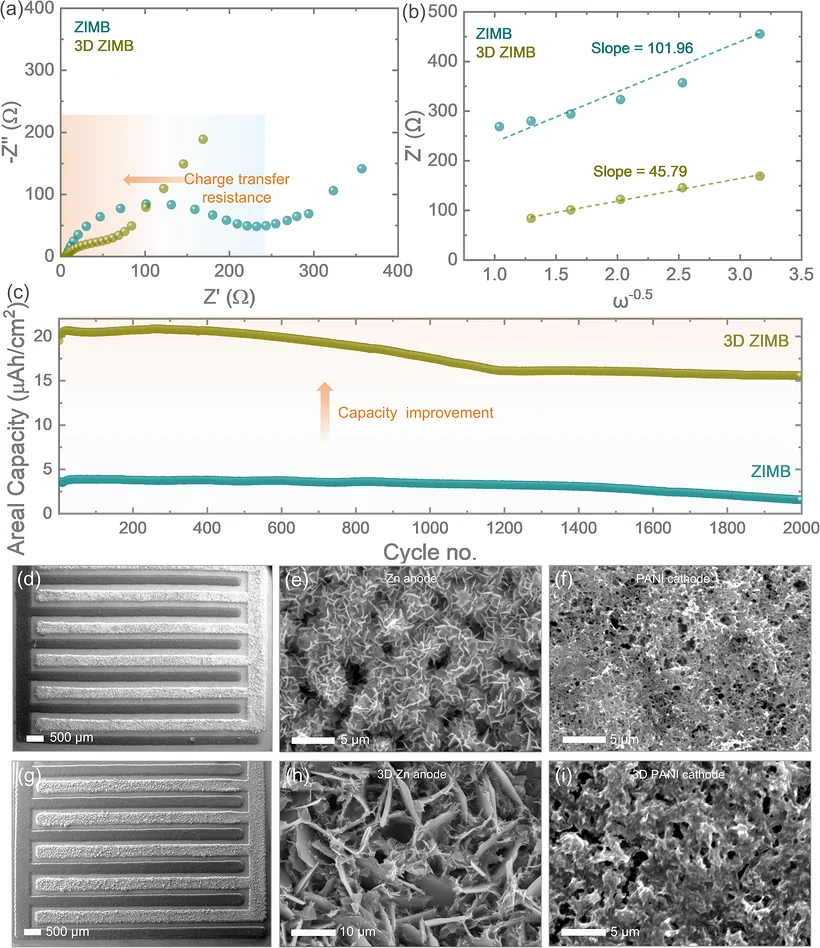
(a) Comparative Nyquist plots of ZIMB and 3D ZIMB, showing a significant reduction in charge‐transfer resistance for the 3D ZIMB. (b) Relative Zn^2^^+^ ion diffusion coefficients of ZIMB and 3D ZIMB, highlighting the improved diffusion kinetics enabled by the 3D Ni scaffolds. (c) Long‐term cycling performance of ZIMB and 3D ZIMB over 2000 cycles. Post‐mortem SEM images of (d–f) ZIMB and (g–i) 3D ZIMB at different magnifications after 2000 cycles.

Electrochemical impedance spectroscopy (EIS) was performed in the frequency range of 10 mHz to 100 kHz with a 10 mV AC voltage amplitude to probe charge‐transfer kinetics and evaluate the influence of 3D porous electrode structures on electrochemical performance. The Nyquist plots (Figure 4a and Figure S9a) reveal that the charge‐transfer resistance of the planar ZIMB is ∼ 226 Ω, which decreases sharply to ∼ 50 Ω in the 3D ZIMB. This substantial reduction confirms that the incorporation of 3D Ni scaffolds effectively lowers internal resistance and significantly enhances charge‐transfer efficiency in the MB system. To further investigate ion‐transport properties, relative Zn^2^^+^ diffusion coefficients were estimated from the linear region of the Nyquist plots using the relations: *Z*′ = (*R_L_* + *R_D_*)  + σω^−0.5^ and $D_{Zn^{2+}}=\frac{R^{2}T^{2}}{2A^{2}n^{4}F^{4}C^{2}\sigma^{2}}=\frac{K}{\sigma^{2}}$; where (*R_L_* + *R_D_*) is the overall resistance (solution and charge transfer resistance), σ is the Warburg coefficient, ω is the angular frequency, *Z*′ is the real impedance component of the Nyquist plot, $D_{Zn^{2+}}$ is the Zn^2^^+^ ion diffusion coefficient (cm^2^ s^−^^1^), R is the molar gas constant (8.314 J K^−^^1^ mol^−^^1^), T is the cell testing temperature in Kelvin (298.13 K), 𝑛 is the number of electrons transferred per electrolyte per monomer unit of PANI, *A* is the active electrode area (cm^2^), 𝐹 is Faraday's constant (96485.3383 C mol^−^^1^), and 𝐶 is the Zn^2^^+^ ion molar concentration used in the electrolyte [24]. Since the device area and electrolyte concentrations during testing are the same, we can consider $k=\frac{R^{2}T^{2}}{2A^{2}n^{4}F^{4}C^{2}}\;$ the same for all the MBs. Therefore, Zn^2^^+^ ion diffusivity is inversely proportional to σ^2^, and lower slopes in the *Z*′ *vs* ω^−0.5^ plots correspond to higher Zn^2^^+^ diffusion coefficients. As shown in Figure 4b and Figure S9b, the planar ZIMB exhibits the highest slope (∼ 101.96), corresponding to the lowest Zn^2^^+^ diffusion coefficient. In contrast, the asymmetric PANI//Zn(3D) MB shows a reduced slope of ∼ 69.83, while the 3D ZIMB achieves the lowest slope of ∼ 45.79. These results confirm that introducing 3D porous Ni scaffolds not only accelerates charge‐transfer kinetics but also markedly improves Zn^2^^+^ ion diffusion, contributing to the superior electrochemical performance of the 3D ZIMBs. Additionally, we investigated the influence of electrolyte amount on the charge transfer resistance and overall electrochemical performance. As shown in Figure S10, the charge transfer resistance of ZIMBs with 100, 300, and 1000 µL of electrolyte is approximately 353, 288, and 226 Ω, respectively. These results indicate that insufficient electrolyte leads to higher charge transfer resistance due to limited ionic transport and incomplete electrode/electrolyte contact. Furthermore, additional long‐term cycling tests were carried out to evaluate the influence of electrolyte amount on capacity retention and cycling stability. As shown in Figure S11, ZIMBs with insufficient electrolyte exhibited lower initial capacity and poorer cycling stability. After 1500 cycles, the devices using 100 µL electrolyte retained only 44.7% of their initial capacity. Although both the 300 and 1000 µL devices demonstrated similar areal capacities up to approximately 1000 cycles, the long‐term stability of the 1000 µL system was significantly better. The ZIMB with 300 µL electrolyte showed a capacity retention of 51.5% after 1500 cycles, whereas those using 1000 µL PVA electrolyte maintained 73.5% of their initial capacity. These results clearly demonstrate that sufficient electrolyte supply improves ionic transport, reduces interfacial resistance, and enhances long‐term electrochemical stability. Therefore, we selected 1000 µL as the optimal electrolyte amount for our MB system. Long‐term cycling stability was further evaluated at an areal current of 1.0 mA cm^−^^2^ (Figure 4c and Figure S12). In the absence of 3D Ni scaffolds, the ZIMB exhibited poor durability, with capacity steadily decreasing during the first 1000 cycles and stabilizing at only ∼ 40% of the initial value thereafter. The asymmetric PANI//Zn(3D) MB maintained ∼85% of its initial capacity for the first 1100 cycles but then suffered a continuous decline, retaining only ∼30% after 2000 cycles. By contrast, the 3D ZIMB displayed excellent cycling stability, with only a slight decrease during the first 1200 cycles before stabilizing in the subsequent 800 cycles. After 2000 cycles, devices incorporating Ni scaffolds consistently retained ∼75% of their initial areal capacity. Moreover, the use of 3D scaffolds substantially enhanced the absolute capacity, increasing from below 5 µAh cm^−^^2^ in planar devices to above 15 µAh cm^−^^2^ in the 3D configuration. Post‐mortem SEM analysis was performed to investigate electrode morphology after extended cycling (Figure 4d–i). The PANI cathodes largely preserved their morphology, although some localized aggregation of polymer chains was observed, likely due to repetitive redox processes. On the Zn anodes, characteristic 2D nanowhiskers were detected, consistent with Zn plating/stripping behavior. Raman spectroscopy after 2000 cycles (Figure S13) further confirmed the stability of the cathode material, with peak positions remaining unchanged relative to the pristine state. Variations in peak intensity were attributed to differences in the relative proportions of –NH–, –NH^+^–, –NH^+^ =, and –N = species, reflecting the dynamic redox transitions of PANI during long‐term cycling. According to Figure S14, the charge transfer resistance of the conventional ZIMB and the 3D ZIMB after cycling is approximately 1600 and 400 Ω, respectively. It is clear that the internal resistance of the 3D MB remains significantly lower than that of the flat ZIMB, indicating that the porous Ni scaffold maintains good structural and electrochemical stability throughout the cycling process. This result suggests that the Ni scaffold continues to provide efficient charge transport and does not undergo severe degradation in the aqueous electrolyte system.

**FIGURE 5:**
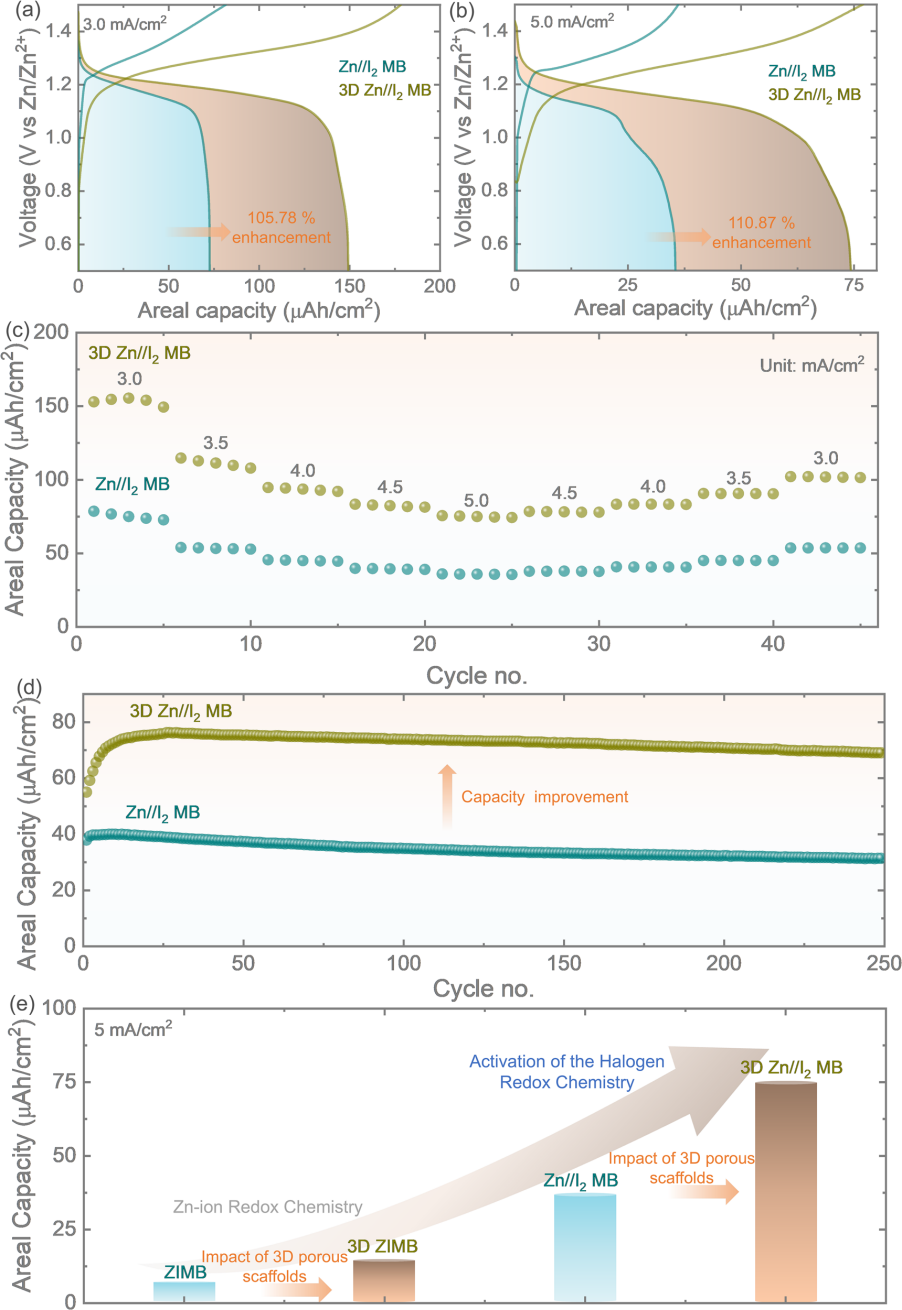
CV curves of Zn//I_2_ and 3D Zn//I_2_ MBs at (a) 3 mA cm^−^^2^ and (b) 5 mA cm^−^^2^. (c) Comparative rate performance of Zn//I_2_ and 3D Zn//I_2_ MBs across areal currents from 3 to 5 mA cm^−^^2^. (d) Long‐term cycling stability of Zn//I_2_ and 3D Zn//I_2_ MBs. (e) Areal capacities of the MBs at a high areal current of 5 mA cm^−^^2^, demonstrating how activation of halogen redox chemistry significantly enhances charge‐storage performance within the same device footprint, while the introduction of 3D porous Ni scaffolds further boosts capacity, highlighting the critical role of electrode architecture.

To further highlight the advantages and uniqueness of the 3D electrode architecture, halogen redox chemistry was activated by simply adding ZnI_2_ salt into the electrolyte (see Experimental Section), as illustrated in Figure 1f. The resulting devices are denoted as Zn//I_2_ MBs (PANI and Zn deposited on Au IDEs) and 3D Zn//I_2_ MBs (PANI and Zn deposited on 3D Ni scaffolds integrated onto Au IDEs). To better understand the optimal concentration of ZnI_2_ in the Zn(CF_3_SO_3_)_2_ electrolyte matrix, we systematically investigated the long‐term cycling stability by varying the ZnI_2_ concentration from 0.1 to 1.0 M, as shown in Figure S15. The effect of different ZnI_2_ concentrations was evaluated through galvanostatic cycling tests under identical conditions. According to Figure S15, the electrolyte containing 0.2 M ZnI_2_ demonstrates the most stable electrochemical performance, showing better capacity retention and overall cycling stability compared to the other concentrations tested. At lower concentrations, the limited amount of active iodine species restricts the redox reaction and reduces the achievable capacity, while higher concentrations may lead to increased side reactions, higher polarization, and faster capacity fading. Therefore, 0.2 M ZnI_2_ provides the best balance between active material availability and electrochemical stability, and was selected as the optimal concentration for incorporation into the electrolyte matrix for all subsequent studies. The electrochemical performance of Zn//I_2_ and 3D Zn//I_2_ MBs was systematically compared at high areal currents (3 – 5 mA cm^−^^2^) to assess the combined effect of halogen redox activation and 3D scaffolds. The GCD curves at 3 and 5 mA cm^−^^2^ (Figure 5a,b) clearly show the superior redox activity of the 3D Zn//I_2_ MBs compared with their planar counterparts, with capacity enhancements of ∼ 105.78% and ∼ 110.87%, respectively. These results confirm that the synergistic effect of halogen redox chemistry and 3D scaffolds substantially improves charge‐storage capability, even under demanding operating conditions. Additional GCD curves at various areal currents are provided in Figure S16. Rate capability tests (Figure 5c) further emphasize these benefits. Across areal currents from 3 to 5 mA cm^−^^2^, the 3D Zn//I_2_ MB consistently delivered higher areal capacities than the planar Zn//I_2_ MB. This improvement highlights the role of the 3D porous Ni scaffold in accelerating Zn^2^^+^ ion diffusion and facilitating electron transport, thereby mitigating kinetic limitations at high currents. Long‐term cycling tests (Figure 5d) also demonstrated long‐term cycling stability. The 3D Zn//I_2_ MB maintained stable capacity retention after 250 cycles, sustaining an areal capacity of ∼ 69.05 µAh cm^−^^2^, whereas the planar Zn//I_2_ MB retained only around ∼ 31.40 µAh cm^−^^2^. These results clearly indicate that the integration of 3D Ni scaffolds not only enhances areal capacity but also significantly improves cycling stability and supports the high‐rate capability of halogen redox chemistry. The Coulombic efficiency of the 3D Zn//I_2_ MB during cycling is shown in Figure S17. The initial Coulombic efficiency was approximately 75%, which can be attributed to the activation process and the initial stabilization of the electrode/electrolyte interface. However, within the first few cycles, the Coulombic efficiency rapidly increased and reached nearly 100%, where it remained stable throughout the subsequent cycling process. This result indicates reversible iodine redox reactions and stability of the 3D Zn//I_2_ MB system during long‐term operation. Post‐cycling SEM images of the electrodes are shown in Figure S18. Most notably, at a high areal current of 5 mA cm^−^^2^, the 3D Zn//I_2_ MB delivers a markedly higher areal capacity than its planar counterpart (Figure 5e). While activation of halogen redox chemistry alone significantly boosts capacity within the same device footprint, the additional integration of 3D porous Ni scaffolds yields even greater improvements, underscoring the critical role of electrode architecture. This dual approach—electrolyte engineering through halogen redox chemistry combined with electrode engineering via 3D scaffolds—offers a highly effective strategy for overcoming the intrinsic limitations of conventional MBs. Collectively, these results demonstrate that coupling halogen redox chemistry with 3D porous scaffolds not only enhances areal capacity and rate performance but also provides durable cycling stability under demanding, high‐current conditions. This combined strategy paves a practical route toward safer, high‐energy, and high‐power MBs, advancing their potential for integration into next‐generation electronic systems.

**FIGURE 6:**
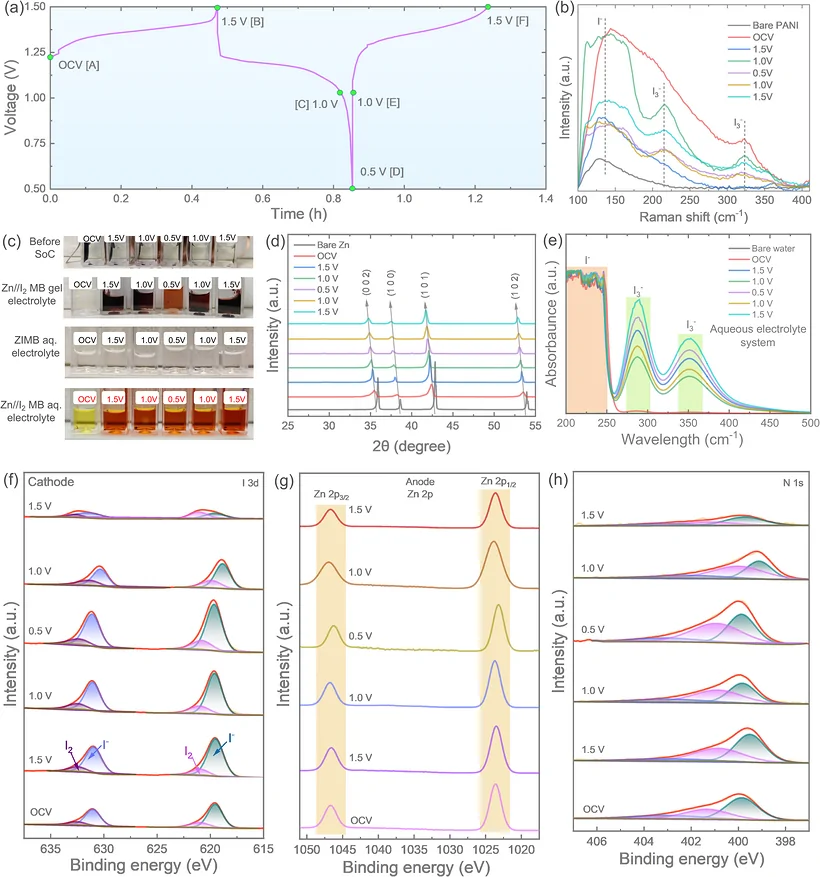
(a) GCD curve showing the specific voltages that align with the State of Charge (SoC) points A–F. (b) Ex situ Raman spectra of bare PANI, PANI electrodes at A (OCV), B (1.5 V), C (1.0 V), D (0.5 V), E (1.0 V), and F (1.5 V) in Zn//I_2_ MBs. (c) Digital images of the electrolytes at SoC, showing the distinct colour evolution during the charge‐discharge process in Zn//I_2_ battery chemistry using both PVA gel electrolyte and aqueous electrolyte, while no obvious colour change is observed in the aqueous electrolyte of Zn‐ion battery chemistry. (d) Ex situ XRD of bare Zn, Zn electrodes at A (OCV), B (1.5 V), C (1.0 V), D (0.5 V), E (1.0 V), and F (1.5 V) in Zn//I_2_ MBs. (e) Ex situ UV‐vis spectra of bare PANI, PANI electrodes at A (OCV), B (1.5 V), C (1.0 V), D (0.5 V), E (1.0 V), and F (1.5 V) in Zn//I_2_ MBs. Ex situ XPS spectra of (f) I 3d, (g) Zn 2p, and (h) N 1s of the electrodes in Zn//I_2_ MBs.

Ex situ investigations were conducted at different state‐of‐charge (SoC) levels to obtain a better understanding of the charge storage mechanism of Zn//I_2_ MBs (Figure 6). We used XPS, Raman, XRD, EDX, and SEM to analyse the corresponding electrodes, and UV‐VIS spectroscopy to characterise the electrolytes at various SoCs. A Zn foil anode was coupled with a PANI cathode electrodeposited on Ti foil for the SoC‐dependent tests. The two electrodes were placed in a cuvette that held an electrolyte solution that included either 2 M Zn(CF_3_SO_3_)_2_ alone and 2 M Zn(CF_3_SO_3_)_3_ with 0.2 M ZnI_2_. The cells underwent charging and discharging processes, maintaining specific voltages that align with the SoC points A–F, as indicated in Figure 6a. Figure 6b shows that Ex situ Raman tests were done at different redox states to help us understand how the PANI‐I_2_ electrode stores charge. The prominent Raman peak, which is around 200∼230 cm^−1^, is the symmetric stretching mode of the tri‐iodide ion (I_3_^−^), and also the peak near 300∼330 cm^−1^ is the symmetric stretching mode of I_3_^−^. These observations are useful for keeping track of how iodine levels change during the galvanostatic charge/discharge process. The I_3_^−^ peak gets less intense as the discharge goes from B to D (1.5 to 0.5 V vs Zn^2+^/Zn). This means that I_3_^−^ has been used up, which means that I_3_^−^ has been completely changed into I^−^. The I_3_^−^ peak comes back during the charging from D to F (0.5 to 1.5 V vs Zn^2+^/Zn). When charged to F (1.5 V), the I_3_^−^ peak has a sharper profile. The redox reaction of PANI‐I_2_ mainly involves the changes between I_3_^−^, and I^−^. This is very different from the way that anions are added and removed in regular PANI electrodes [25]. The digital images recorded at SoC for ZIMBs and Zn//I_2_ MBs in both aqueous electrolyte and PVA gel electrolyte (Figures 6c) reveal a distinct and reversible colour transition of the electrolyte, evolving from transparent yellow to deep orange, then to light orange, and finally returning to deep orange during the charge–discharge process in Zn//I_2_ battery chemistry. Notably, the PVA gel electrolyte exhibits the same colour evolution as observed in the aqueous electrolyte, indicating that a similar redox reaction mechanism occurs in both systems as like aq. System without PVA matrix. Consistent with this observation, similar characteristic peak changes are also observed in the UV‐vis spectra (Figure S19), although the spectral response in the PVA electrolyte is slightly weaker. This reduced absorbance is mainly attributed to the higher viscosity of the gel electrolyte, which slows ion diffusion and reduces the intensity of the spectroscopic response. This colour evolution is characteristic of the reversible electrochemical conversion between I^−^ and I_3_^−^ species, providing visual evidence of the halogen redox process [26]. In contrast, no observable colour change is detected in the electrolyte of ZIMBs without ZnI_2_ at different SoC, as expected, confirming that the colour transition originates exclusively from the presence of iodine species. However, the main reason for performing the Ex situ characterizations using aqueous electrolytes was to obtain clearer and more reliable characterization signals from the electrodes at different states of charge. In practical experiments, removing the PVA gel electrolyte from the electrode surface without disturbing the active materials is quite challenging, and residual PVA can significantly interfere with surface‐sensitive measurements such as XPS and XRD, as well as weaken the analytical signals. Therefore, aqueous electrolyte systems were primarily used to facilitate accurate mechanistic characterization of the electrode reactions (see further).

Figure 6d shows the XRD of the Zn anodes at different SoC. The UV‐VIS spectra illustrated in Figure 6e validate the existence of I^−^ across all conditions, encompassing the pristine electrode, open‐circuit voltage (OCV), and SoC points A (OCV, 1.19 V), B (1.5 V), C (1 V), D (0.5 V), E (1 V), and F (1.5 V). As the cell is charged from A (1.19 V) to higher potentials (e.g., B at 1.5 V), distinct I_3_^−^ peaks appear around ∼ 286 and ∼ 350 nm in the UV‐VIS spectrum [25]. The observed spectral evolution indicates the oxidation of I^−^ ions in the PANI polyelectrolyte to I_2_, which then interacts with remaining I^−^ to produce soluble I_3_^−^, resulting in the yellow colouration of the electrolyte. By comparison, there is no peaks in the corresponding area in ZIMBs, as shown in Figure S20. To better understand this redox behavior, XPS analysis was done on the electrodes at SoC levels (Figure 6f–h). The I 3d spectra that go with the PANI cathode are shown in Figure 6f. At OCV, there are clear peaks for I 3d_5_/_2_ and I 3d_3_/_2_ at about 631.5 and 619.9 eV, respectively. When the battery is fully discharged at point D (0.5 V), the I 3d peaks move to higher binding energies. The deconvoluted peaks are at about 632.3 and 630.1 eV (I 3d_5_/_2_) and 619.97 and 621.80 eV (I 3d_3_/_2_). This indicates a partial reduction of I_2_ to I^−^ and the existence of both oxidation states [27, 28]. In the fully charged state (point B and F, 1.5 V), the spectra reveal a prominent I^−^ peak (∼ 630.36 eV) accompanied by a lesser I_2_ peak (∼ 631.55 eV), indicating the oxidation of I^−^ to I_2_. Upon returning to the fully discharged state at point D (0.5 V), the I 3d peaks shift back to higher binding energies, with a dominant I_2_ peak at approximately 632.50 eV and a minor I^−^ peak at around 630.30 eV, indicating significant conversion of I^−^ back to solid I_2_ [27, 28]. The results confirm the dynamic coexistence and transformation of I^−^ and I_2_ species during the charge‐discharge cycle. The redox mechanism described involves I^−^ ions in the PANI matrix, which lose electrons during the charging process to form solid I_2_ (2I^−^ → I_2_ + 2e^−^). The reaction I_2_ + I^−^ → I_3_^−^ changes solid I_2_ into I_3_^−^. The Zn 2p XPS spectra for the Zn anode at different charge levels (Figure 6g) also show consistent signals for both Zn 2p_3_/_2_ (∼1047 eV) and Zn 2p_1_/_2_ (∼1023 eV). This shows that Zn is present in all charge states (A–F). The SoC analysis also looks at the ZIMB cell (Figure S21), which is built the same way but doesn't have ZnI_2_ in the electrolyte. These tests help us understand the 2e^−^ phenomena better. The colour of the electrolyte without ZnI_2_ stayed the same and almost transparent during the whole test and at the end, just like it did at the beginning or during the open‐circuit voltage phase (Figure 6c). The UV‐VIS data for the Zn(CF_3_SO_3_)_2_ (2 M) electrolyte without iodine clearly shows that there are no I^−^ or I_3_^−^ peaks, portrays in supplementary (Figure S20b). To examine the structural and compositional changes that happen during cycling in more detail, we used SEM imaging and EDX mapping on the electrodes at different SoC levels (Figure S21). Figure 6h shows how the valence of nitrogen atoms in PANI‐I_2_ changes in different redox states. The electrode's N1s signal has two peaks, one at about 399.6 eV and the other at about 401.5 eV. These peaks are linked to non‐protonated amine −NH− (reductive state) and protonated imine −NH^+^ = (bipolaron, oxidised state) [27]. At OCV state A, the calculated content of −NH− and −NH^+^ = aligned with findings from other studies on polyiodide doped PANI [28]. Given the significant changes in the valence state content of nitrogen, which can be attributed to the doping and de‐doping processes of anions, we propose that in this study, following the transformation of I_3_^−^ into I^−^, only a small fraction of I^−^ undergoes de‐doping, while the majority remains confined within the PANI chain. The SEM mapping (Figures S20 and S21) reveals a significant increase in the intensity of Zn elements at the discharged state, suggesting the adsorption of Zn^2+^ into PANI‐I_2_. The C 1s XPS spectra (Figure S20d) show that the PANI cathode changes in different ways depending on the potential. This shows that the polymer goes through reversible redox (doping‐dedoping) processes while the battery is working. The increased presence of C–N/C = N and higher‐binding‐energy components during charging suggests oxidation and the creation of polarons and bipolarons. Their partial recovery during discharge confirms that the process can be reversed. This behavior shows that PANI can efficiently compensate for charge at the interface in the Zn(CF_3_SO_3_)_2_+ZnI_2_ electrolyte system. The deconvoluted peaks are identified as approximately 284.6–284.8 eV, corresponding to C–C / C═C (benzenoid backbone of PANI); around 285.8–286.2 eV, relating to C–N / C═N (quinoid units, oxidised PANI); and approximately 287.8–288.6 eV, associated with C–N^+^ / C═O–like species (protonated or highly oxidised states, exhibiting strong polaron/bipolaron character). The EDS mapping of the PANI cathode at state of charge points (Figure S21a–g) confirms that carbon, nitrogen, iodine, and zinc are all present. In contrast, the SEM images and EDX mapping of the Zn anodes at OCV SoC point A (1.19 V) (Figure S21h) show a relatively flat surface, exhibiting a uniform elemental distribution. At elevated states of charge, the Zn anodes display the formation of irregular spherical particles or flakes, as illustrated in Figure S21i–n. The observed features can be attributed to the migration of polyiodide species, which play a role in localised corrosion on the Zn surface [29]. The EDS mapping of the Zn anode at the state of charge point F (Figure S21n) provides additional confirmation of the distribution of Zn and I, thereby supporting the existence of iodine‐related corrosion products.

**FIGURE 7:**
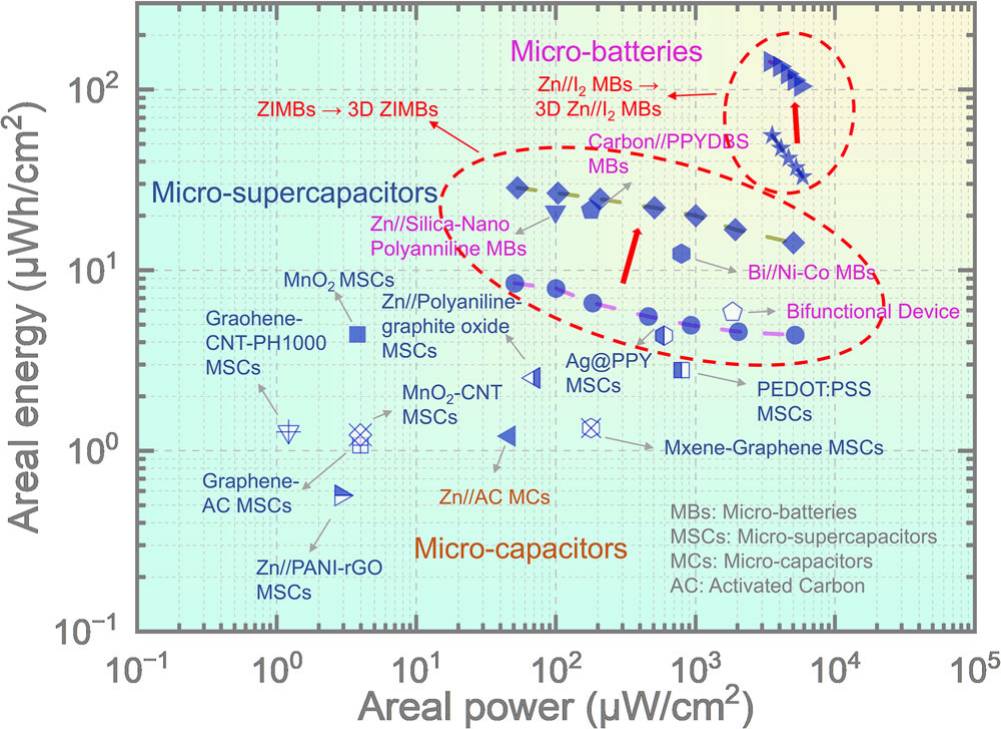
Ragone plot comparing the areal energy and areal power of the 3D ZIMB and 3D Zn//I_2_ MBs, highlighting how the combination of halogen redox chemistry and 3D structuring enhances performance relative to reported high‐performance MBs: Zn//Silica‐Nano Polyaniline [30], Carbon//PPYDBS [31], Bi//Ni‐Co [32], micro‐capacitors (MCs): Zn//AC [33], and micro‐supercapacitors (MSCs): MnO2‐CNTs [34], Graphene/CNT/cross‐linked PH1000 film(GCP) [35], Zn//Polyaniline‐graphite oxide [36], Ag@PPY [37], PEDOT:PSS [38], Mxene‐Graphene [39], Graphene‐AC [40], Zn//PANI‐rGO [41], MnO_2_ [42].

The areal energy of the devices was further evaluated at different areal currents. At 50 µA cm^−^^2^, the ZIMB and 3D ZIMB delivered areal energies of 8.43 and 28.56 µWh cm^−^^2^, respectively, while at 1 mA cm^−^^2^, these values were 4.94 and 20.02 µWh cm^−^^2^. The corresponding areal power outputs of the 3D ZIMBs were 53.43 µW cm^−^^2^ (at 28.56 µWh cm^−^^2^), 207.64 µW cm^−^^2^ (at 24.69 µWh cm^−^^2^), 1015.09 µW cm^−^^2^ (at 20.02 µWh cm^−^^2^), and 5090.22 µW cm^−^^2^ (at 14.14 µWh cm^−^^2^), compared with 51.01 µW cm^−^^2^ (at 8.43 µWh cm^−^^2^), 184.54 µW cm^−^^2^ (at 6.56 µWh cm^−^^2^), 935.61 µW cm^−^^2^ (at 4.94 µWh cm^−^^2^), and 5221.73 µW cm^−^^2^ (at 4.35 µWh cm^−^^2^) for ZIMBs. These results confirm that the incorporation of Ni scaffolds significantly enhances areal energy while maintaining comparable power densities. As anticipated, performance was further improved upon introducing halogen redox chemistry: at 3 mA cm^−^^2^, Zn//I_2_ and 3D Zn//I_2_ MBs delivered areal energies of 55.63 and 142.53 µWh cm^−^^2^, with corresponding areal powers of 3567.53 and 3443.59 µW cm^−^^2^. These results clearly demonstrate that integrating halogen redox chemistry with 3D electrode architectures produces a step‐change improvement in energy density without sacrificing power output. For benchmarking, the Ragone metrics of our devices were compared with previously reported microscale energy storage systems (Figure 7). The results clearly show that our MBs outperform many state‐of‐the‐art devices, delivering higher performance than reported high‐performance MBs: Zn//Silica‐Nano Polyaniline [30], Carbon//PPYDBS [31], Bi//Ni‐Co [32], micro‐capacitors (MCs): Zn//AC [33], and micro‐supercapacitors (MSCs): MnO_2_‐CNTs [34], Graphene/CNT/cross‐linked PH1000 film(GCP) [35], Zn//Polyaniline‐graphite oxide [36], Ag@PPY [37], PEDOT:PSS [38], Mxene‐Graphene [39], Graphene‐AC [40], Zn//PANI‐rGO [41], MnO_2_ [42], respectively. These results confirm that the dual strategy of 3D structuring and halogen redox chemistry provides a robust pathway toward safer, high‐energy, and high‐power MBs, offering strong potential for integration into next‐generation electronic systems.

## 3 Conclusion

In this work, we have demonstrated a dual strategy combining 3D porous Ni scaffolds with halogen redox chemistry to realize high‐performance Zn‐based microbatteries. The introduction of 3D scaffolds through the dynamic hydrogen bubble template (DHBT) method enabled higher active material loading, improved ion/electron transport, and reduced charge‐transfer resistance, leading to substantial enhancements in areal capacity, rate capability, and long‐term cycling stability. Compared with planar ZIMBs, the 3D ZIMBs delivered nearly two‐fold improvements in areal capacity and superior retention of 96.04% after extended high‐rate cycling, underscoring the effectiveness of 3D structuring in overcoming the limitations of conventional micro‐battery designs. Beyond geometric optimization, we further exploited electrolyte engineering by introducing ZnI_2_ into the gel electrolyte to activate halogen redox chemistry. This modification provided an additional redox couple (I^−^/I_3_^−^), significantly enhancing charge‐storage capacity without altering electrode composition or device footprint. Most strikingly, the 3D Zn//I_2_ MB achieved an areal energy of 142.53 µWh cm^−^^2^ at 3 mA cm^−^^2^, far exceeding that of planar Zn//I_2_ MBs (55.63 µWh cm^−^^2^), while maintaining comparable power outputs (∼3.5 mW cm^−^^2^). This synergy between 3D scaffolding and halogen redox chemistry resulted in a step‐change improvement in energy density and stable high‐rate performance. Benchmarking against reported micro‐scale storage technologies further confirms the competitiveness of our approach. The 3D Zn//I_2_ MBs not only surpass many state‐of‐the‐art Zn‐based microbatteries but also rival or outperform advanced micro‐supercapacitors in terms of power density, while simultaneously delivering much higher energy densities. This positions our devices among the most efficient aqueous micro‐power sources reported to date. Overall, this study establishes that integrating 3D electrode architectures with halogen redox chemistry provides a powerful and practical pathway toward safer, high‐energy, and high‐power Zn‐based microbatteries. The demonstrated improvements in areal capacity, energy density, rate capability, and durability highlight the promise of this strategy for powering next‐generation wearable, implantable, and on‐chip electronic systems.

## Author Contributions

**Yijia Zhu**: conceptualization, methodology, data curation, formal analysis, validation, investigation, visualization, writing original draft, writing review and editing. **Monojit Mondal**: data curation, formal analysis, validation, investigation, writing original draft, writing review and editing, visualization. **Xiaopeng Liu**: data curation, formal analysis, validation, writing review and editing. **Nibagani Naresh**: data curation, formal analysis, writing review and editing. **Firoz Alam**: data curation, writing review and editing. **Mingqing Wang**: writing review and editing. **Buddha Deka Boruah**: conceptualization, supervision, resources, project administration, writing review and editing, writing original draft, funding acquisition, visualization.

## Conflicts of Interest

The authors declare no conflicts of interest.

## Supporting information




**Supporting File**: advs75965‐supp‐0001‐suppMat.pdf.

## Data Availability

The datasets generated and/or analyzed during the current study are available from the corresponding author on reasonable request.
