# Supplementary material for: Synergistic 3D Porous Architectures and Halogen Redox Chemistry for High‐Energy and High‐Power Microbatteries
Source: Adv Sci (Weinh). 2026 Jun 2;13(48):e75956. doi: 10.1002/advs.75956 (PMC13336436; doi:10.1002/advs.75956)
Supplement: Supplementary file 1 [file ADVS-13-e75956-s001.pdf]

Synergistic 3D Porous Architectures and Halogen Redox Chemistry for High-Energy and High-Power Microbatteries

Yijia Zhu,[1] Monojit Mondal,[1] Xiaopeng Liu,[1] Nibagani Naresh,[1] Firoz Alam,[2] Mingqing Wang,[1] Buddha Deka Boruah[1,*]

[1]Institute for Materials Discovery, University College London, London WC1E 7JE, United Kingdom

[2]Department of Electronic and Electrical Engineering, University College London, London, WC1E 6BT, UK

Experimental Section

Materials: Aniline(Sigma-Aldrich), Sulfric Acid(H_2SO_4, Fisher Bioreagents), Sodium Sulfate Anhydrous(Na_2SO_4, Fisher Bioreagents), Zinc Sulfate Heptahydrate ($ZnSO_4 \cdot 7H_2O$, Thermo Scientific), Boric Acid (Sigma-Aldrich), Nickel Chloride ($NiCl_2 \cdot 6H_2O$, Scientific Laboratory Supplies), Ammonium Chloride(NH_4Cl, Fluoro Chem), Polyvinyl alcohol (PVA, Sigma-Aldrich), Zinc Trifluoromethanesulfonate($Zn(CF_3SO_3)_2$, Fluoro Chem).

Preparation of polyaniline (PANI) cathode and Zn anode: To prepare the electrolyte for PANI deposition, aniline was added to 1 M H_2SO_4 aqueous solution. Separately, a Zn deposition electrolyte was prepared by dissolving 12.5 g Na_2SO_4, 22.3 g $ZnSO_4 \cdot 7H_2O$, and 2 g boric acid in 91 mL deionized (DI) water.

Electrodeposition was performed using a three-electrode configuration, with a commercial gold-patterned chip as the working electrode (held by a platinum clip), an Ag/AgCl electrode as the reference, and a platinum wire as the counter electrode. PANI was electrodeposited at a constant potential of 0.85 V for 30 seconds. Subsequently, Zn was deposited onto the electrode at a constant current of -40 mA for 8 seconds.

Preparation of 3D Ni Zn (NZn) anode: To fabricate the 3D Ni scaffold, an aqueous solution containing 0.2 M $NiCl_2 \cdot 6H_2O$ and 2 M NH_4Cl was used. Electrodeposition was carried out in a two-electrode system with a nickel foam as the counter electrode, applying a constant current density of -2.5 A/cm^2. Deposition times were varied to assess the effect of scaffold thickness. Subsequently, Zn was deposited onto the 3D Ni scaffold using the same procedure described previously, with varying durations to study the influence of Zn loading.

Preparation of PVA gel electrolyte: For ZIMB tests, 1 g of poly(vinyl alcohol) (PVA) powder was dissolved in 10 mL of deionized (DI) water at 85 °C under continuous stirring until a clear and homogeneous solution was obtained. Subsequently, 10.9 g of $Zn(CF_3SO_3)_2$ was gradually added to the hot solution to achieve a final concentration of 2 M, followed by stirring until complete dissolution and gel formation. For Zn//I_2 MB tests, a transparent PVA gel electrolyte was prepared by dissolving 1 g of PVA in 10 g of distilled water in an oil bath at 85 °C for 1 h, yielding a uniform and transparent gel. Afterward, $Zn(CF_3SO_3)_2$ (2 M) and ZnI_2 (0.2 M) were added directly into the gel, and the mixture was maintained at 85 °C under stirring for an additional 2 h to obtain a homogeneous and transparent Zn//I_2 gel electrolyte.

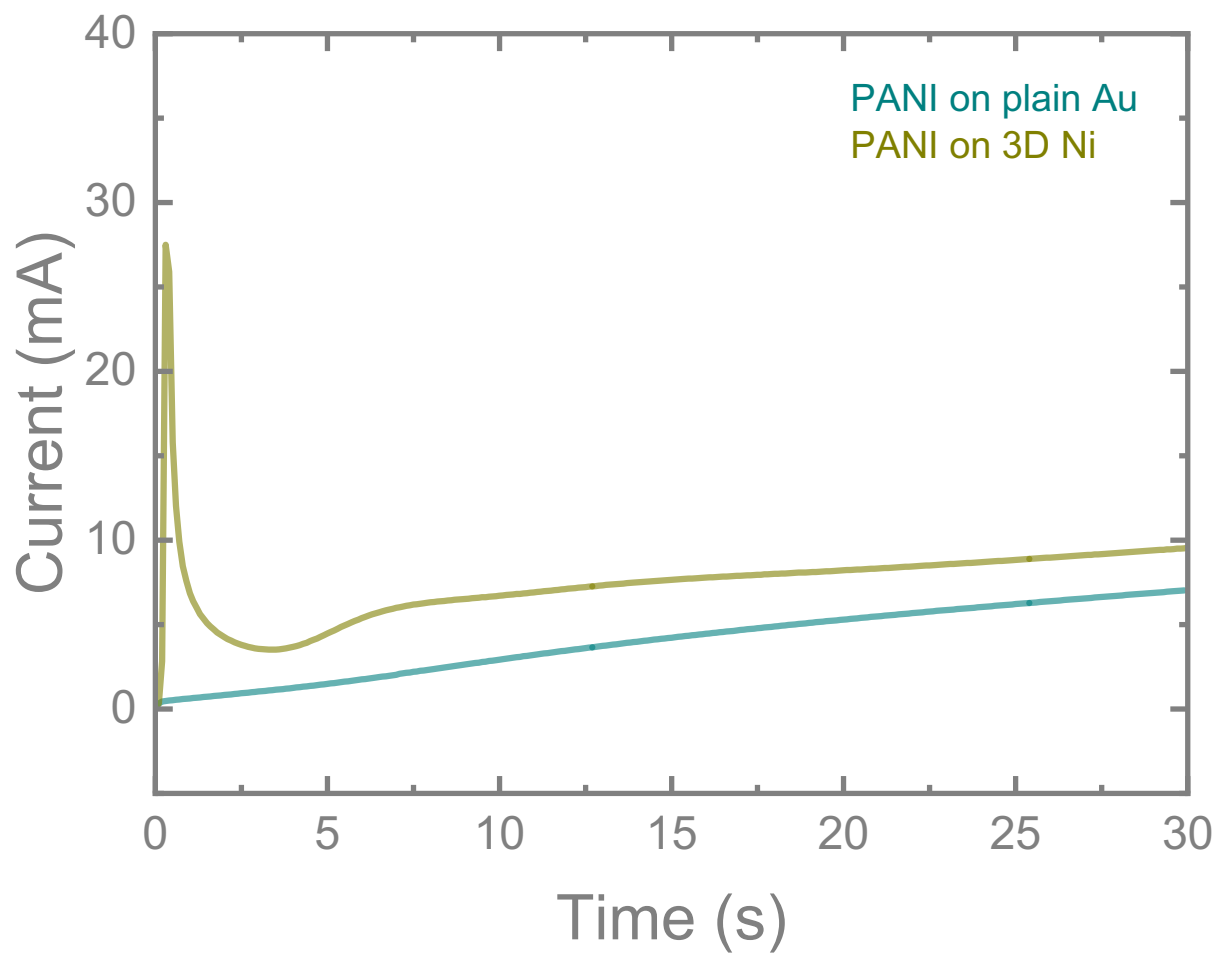

Figure S1. *Electrodeposition curves of PANI on plain Au current collector and 3D porous Ni scaffold.*

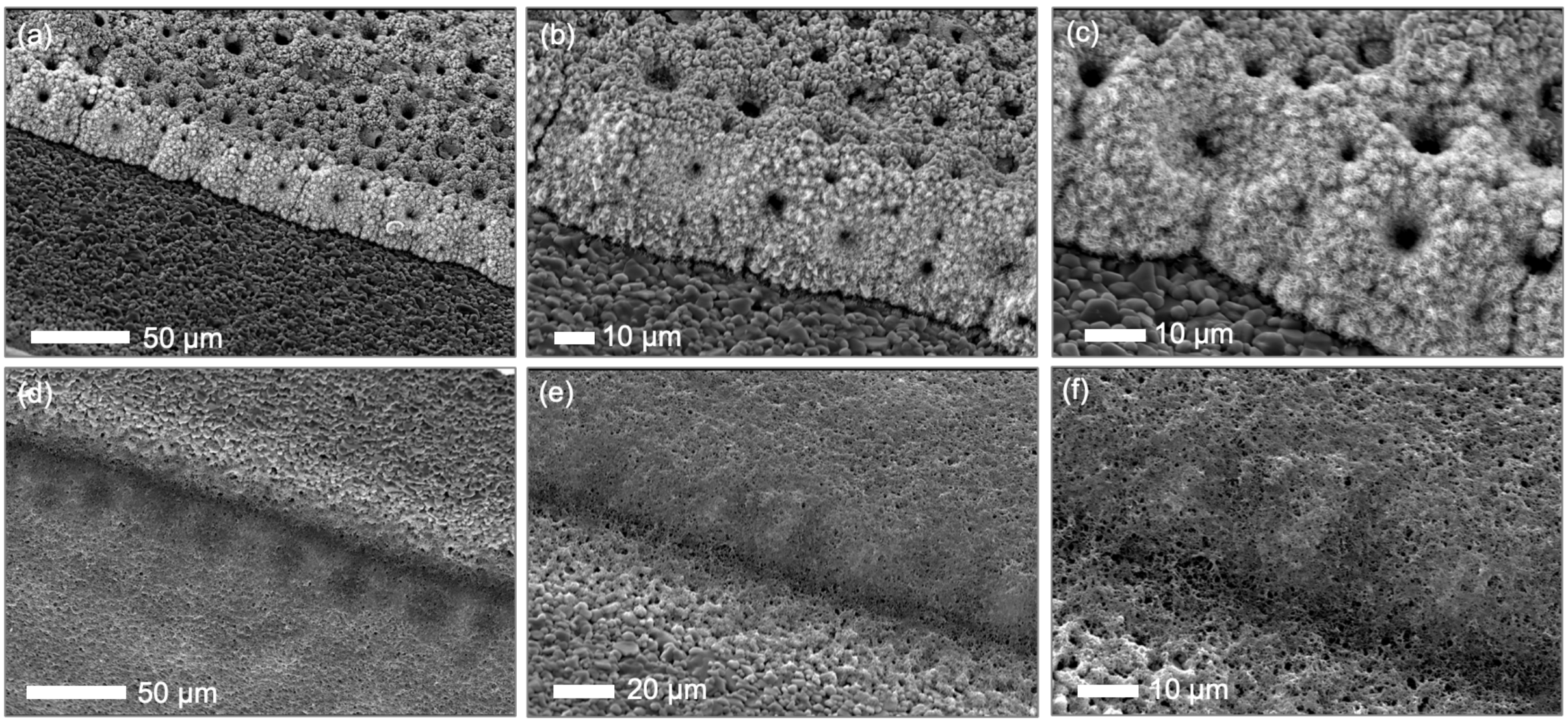

Figure S2. *Cross-sectional SEM images of (a–c) Zn and (d–f) PANI deposited onto the 3D porous Ni scaffold.*

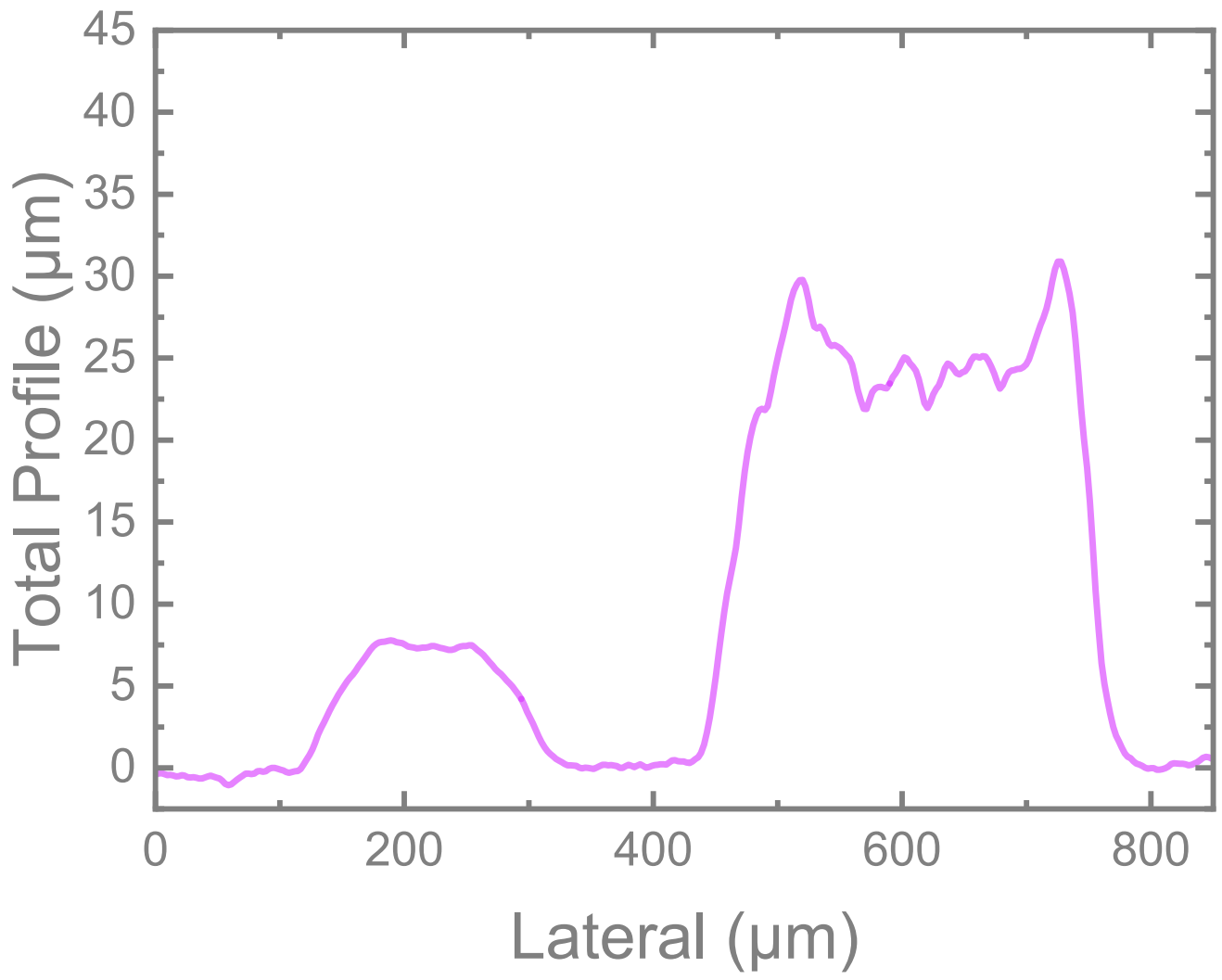

Figure S3. *Profilometry curve of PANI//3D Zn MBs.*

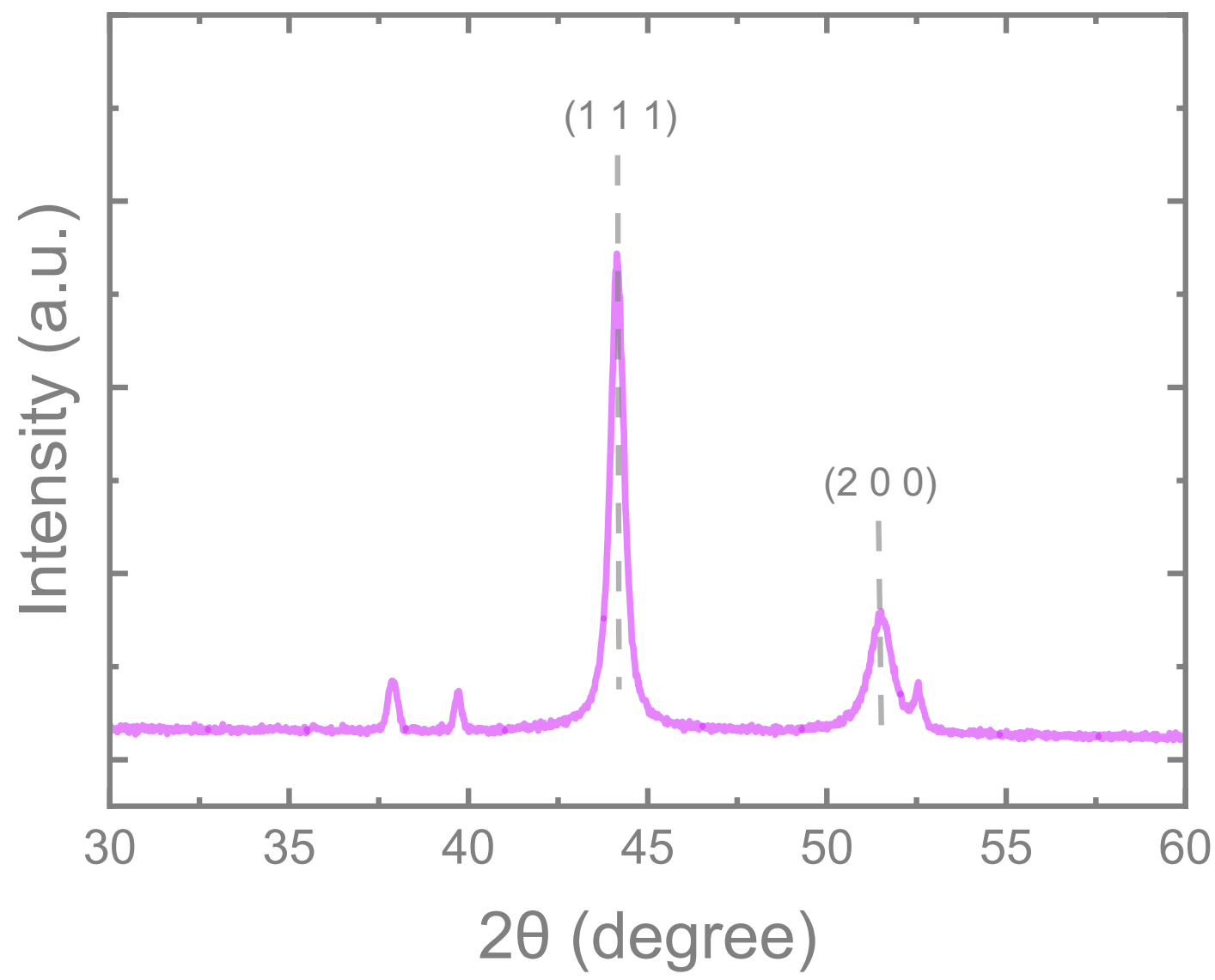

Figure S4. *XRD curve of Ni scaffold on Ti substrate.*

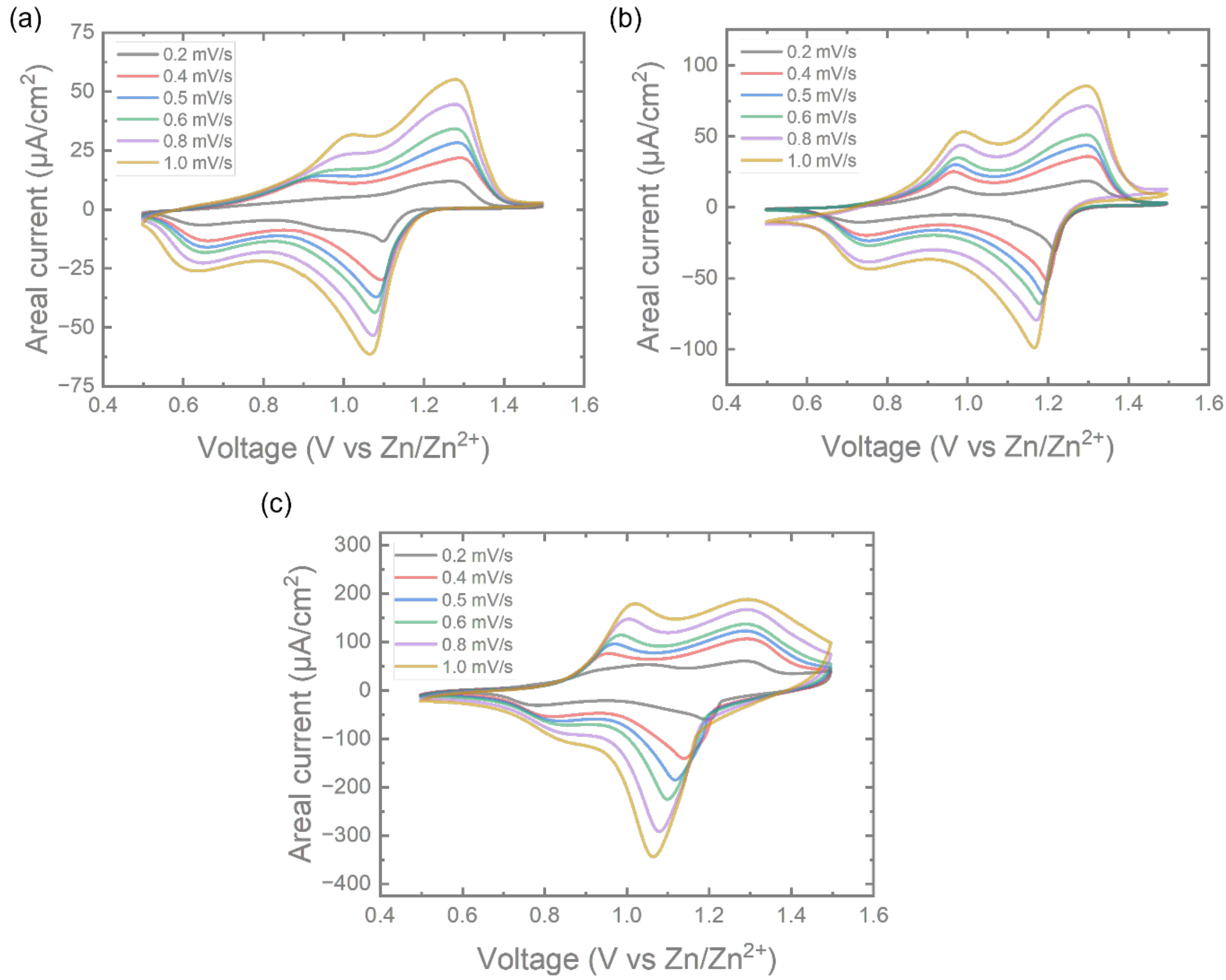

Figure S5. *CV curves of (a) PANI//Zn, (b) PANI//3D Zn, and (c) 3D PANI//3D Zn MBs at different scan rates.*

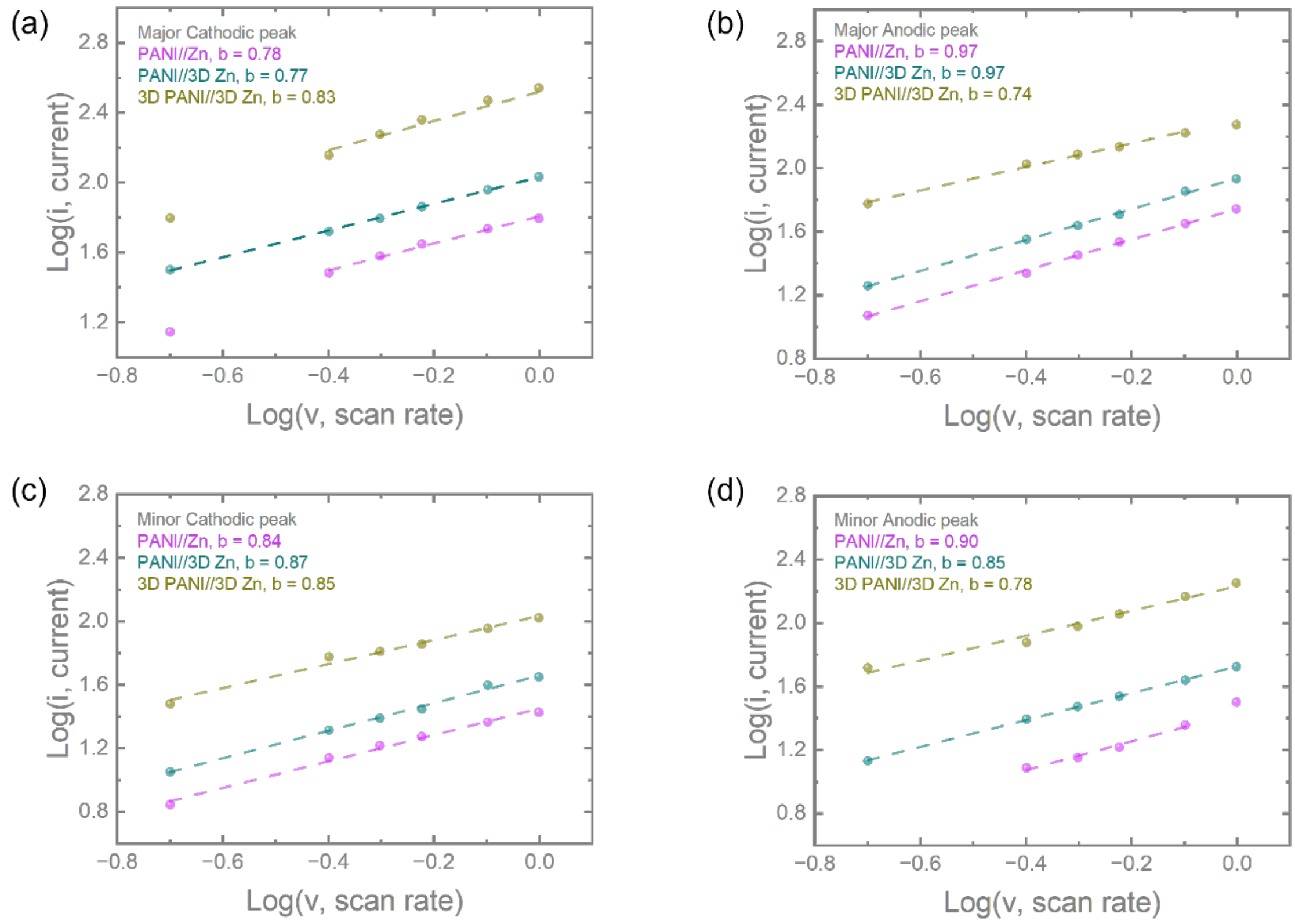

Figure S6. *b values calculations of PANI//Zn, PANI//3D Zn, and 3D PANI//3D Zn MBs from (a) major cathodic peaks, (b) major anodic peaks, (c) minor cathodic peaks, and (d) minor anodic peaks.*

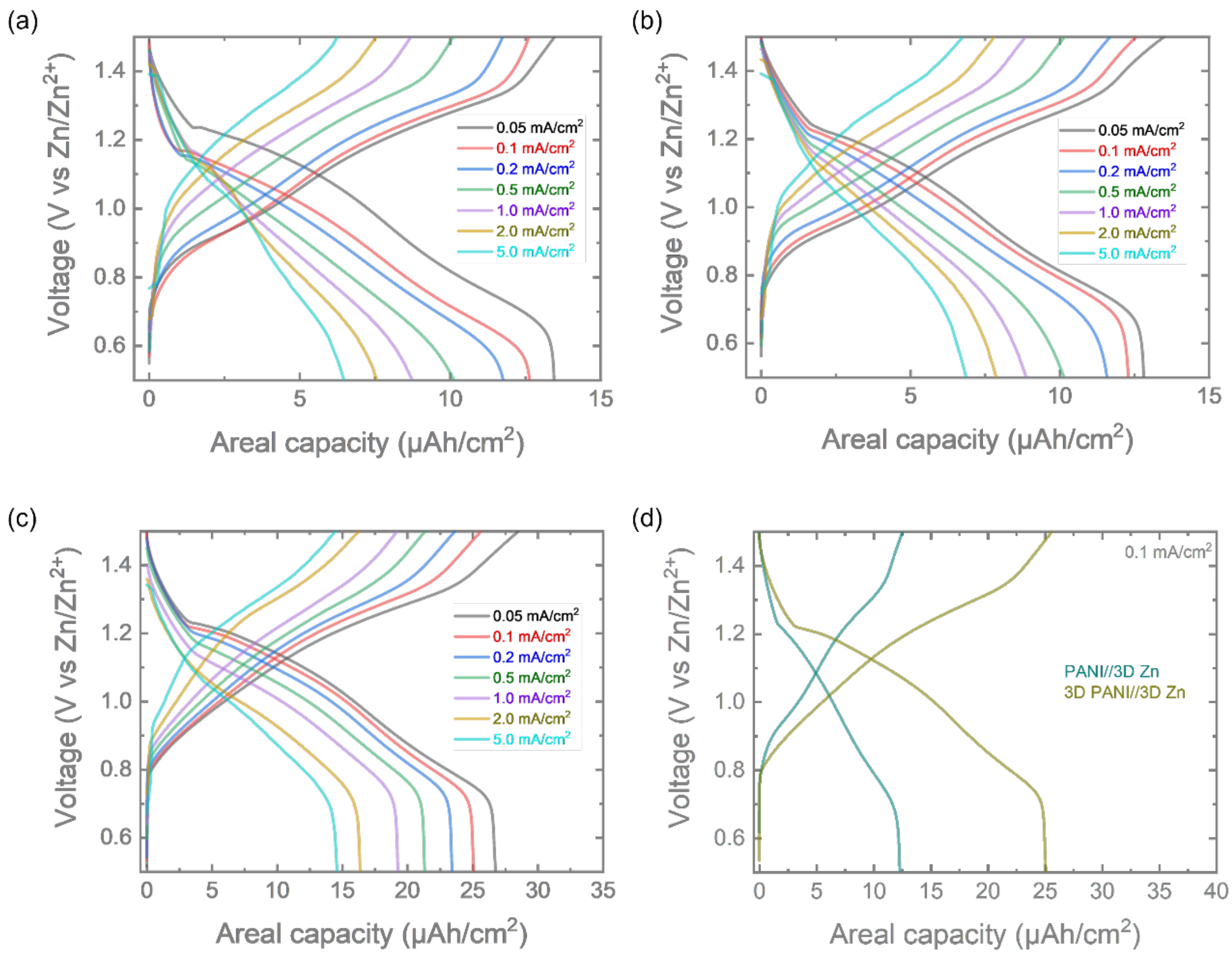

Figure S7. *GCD curves of (a) PANI//Zn, (b) PANI//Zn(3D), and (c) 3D PANI//Zn MBs at different areal currents, and the comparative GCD curves between PANI//Zn(3D) MB and 3D ZIMB at the areal current of 0.1 mA/cm^2.*

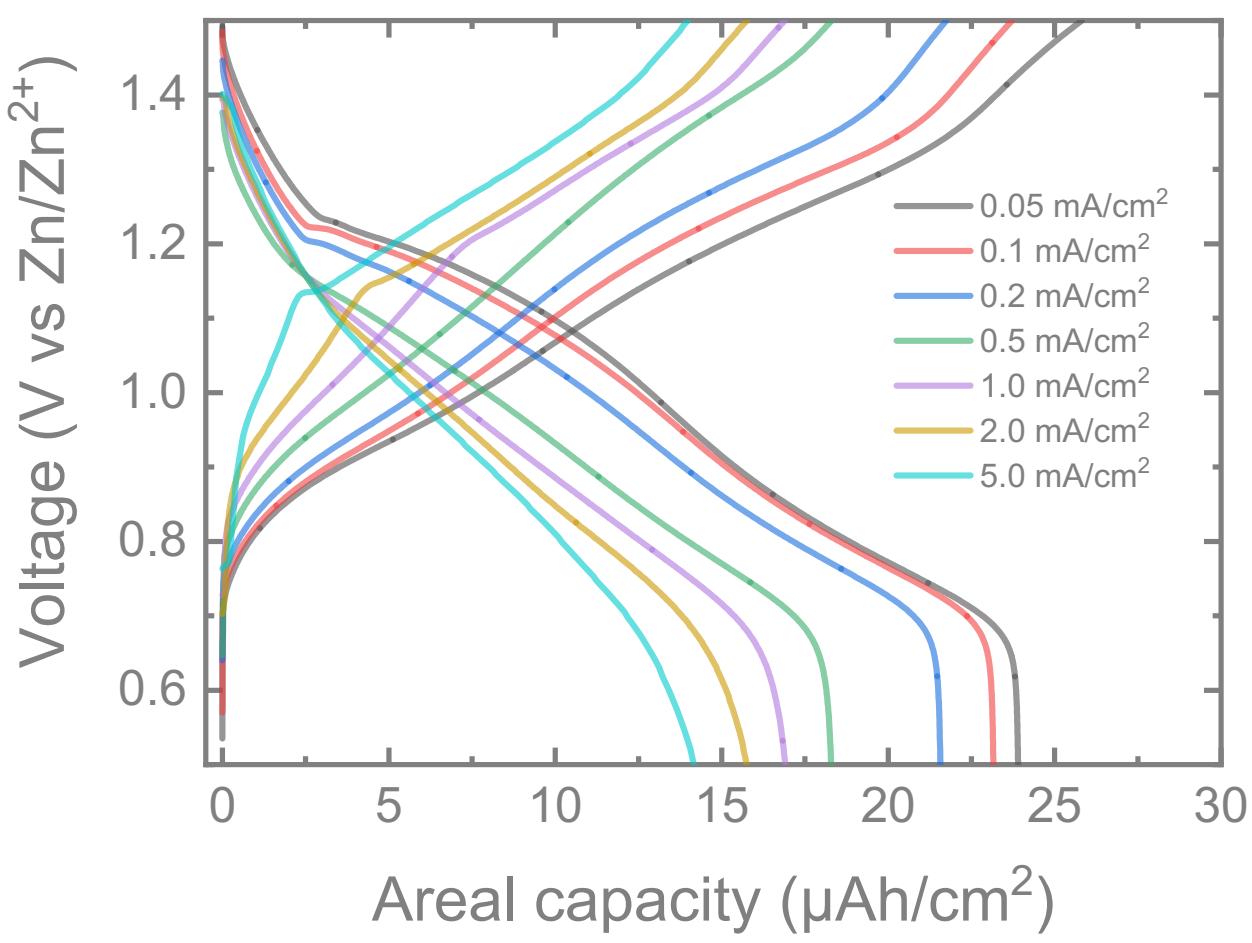

Figure S8. *GCD curves of PANI//Zn(3D) MBs with 20s Ni scaffold on the cathode side at different areal currents.*

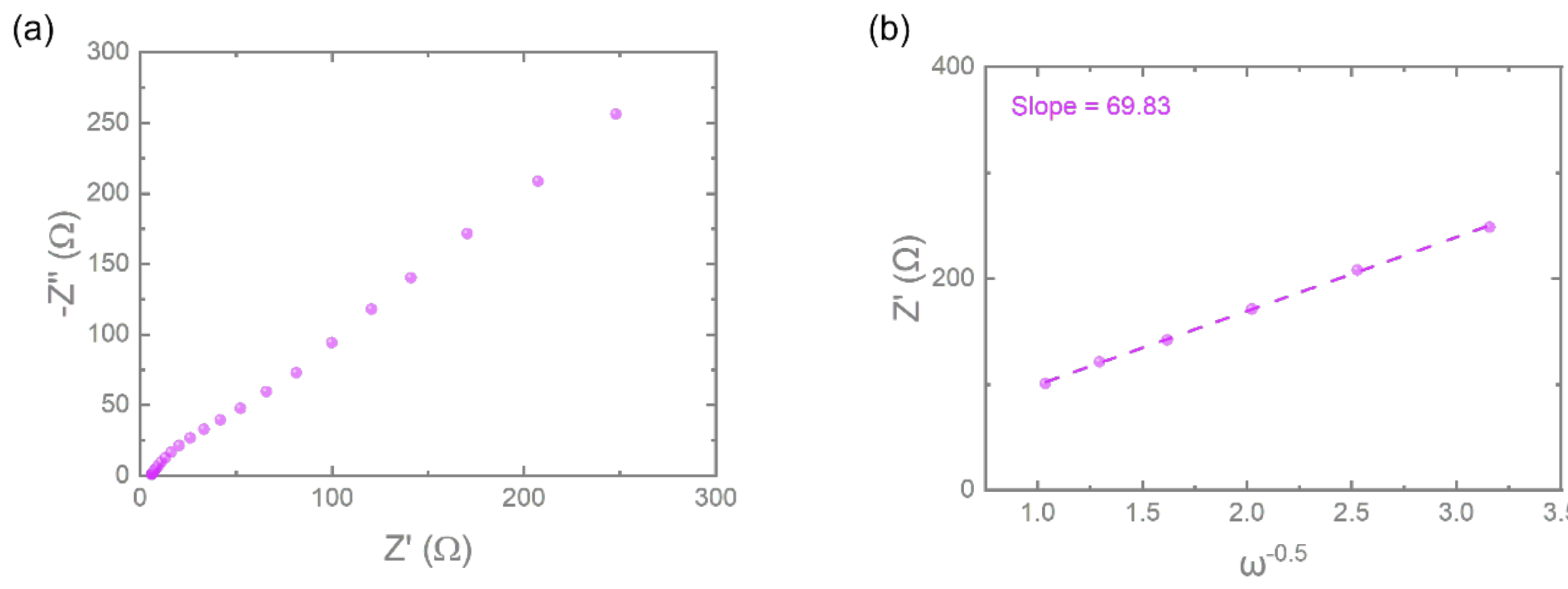

Figure S9. *(a) Nyquist plot and (b) coefficient calculation curve of PANI//3D Zn MBs.*

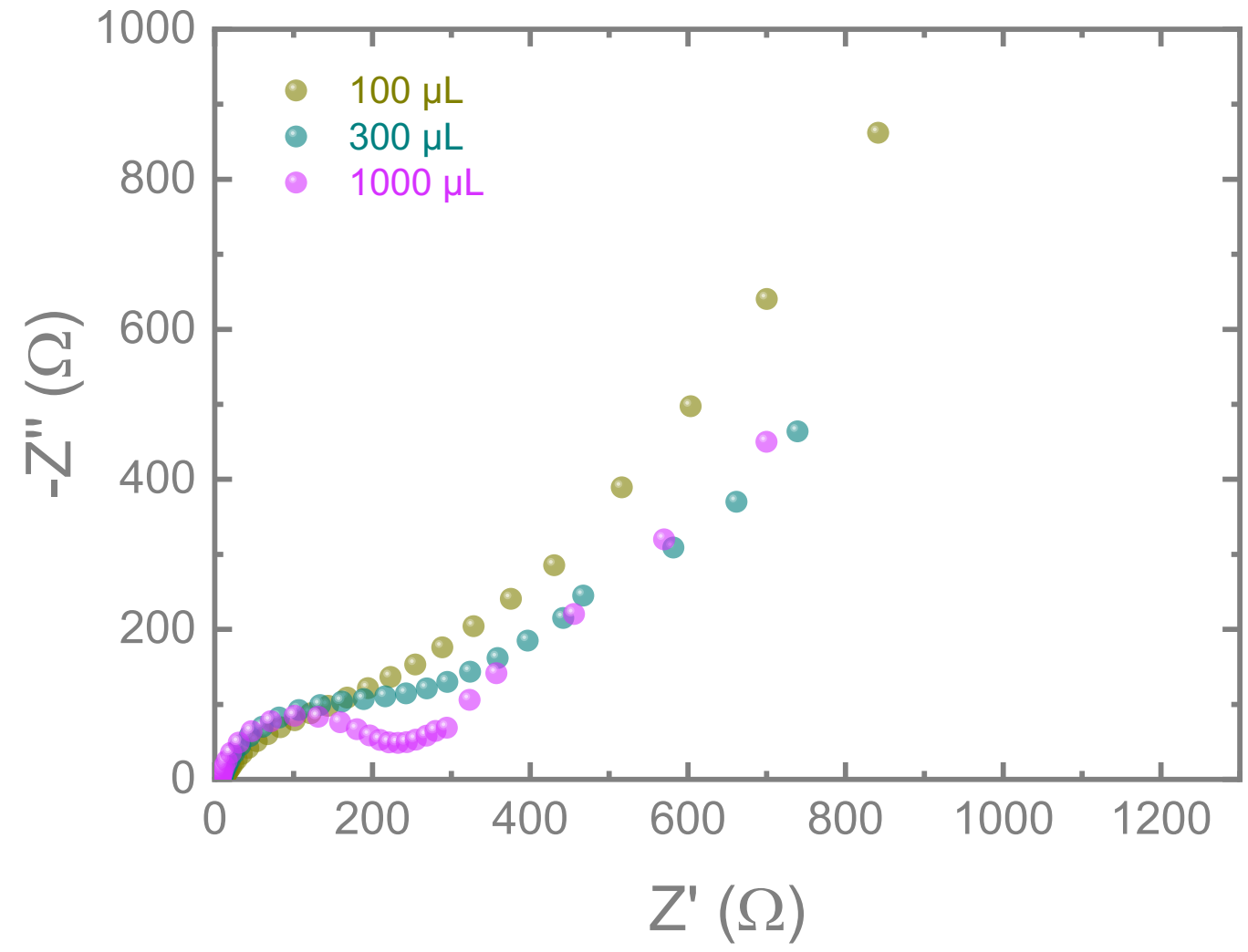

Figure S10. *EIS curves of ZIMBs in 100, 300, and 1000 µL electrolyte.*

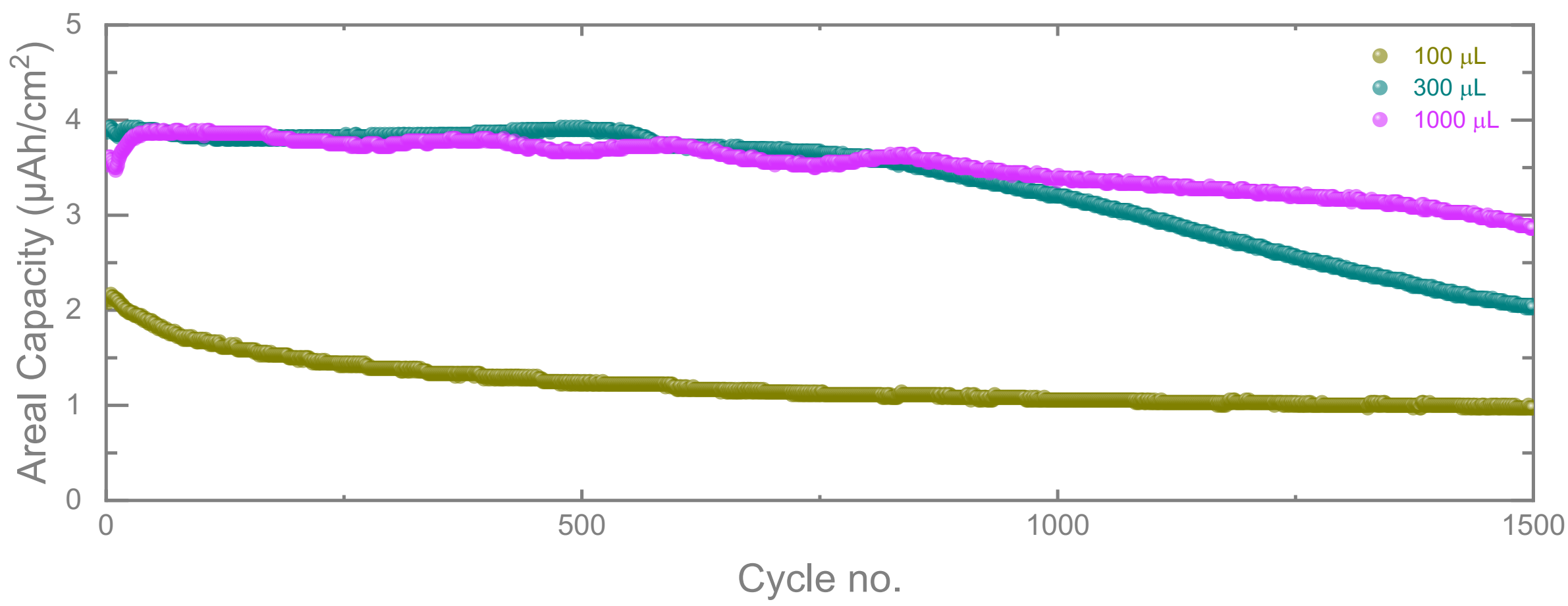

Figure S11. *Long cycling curves of ZIMBs in 100, 300, and 1000 µL electrolyte.*

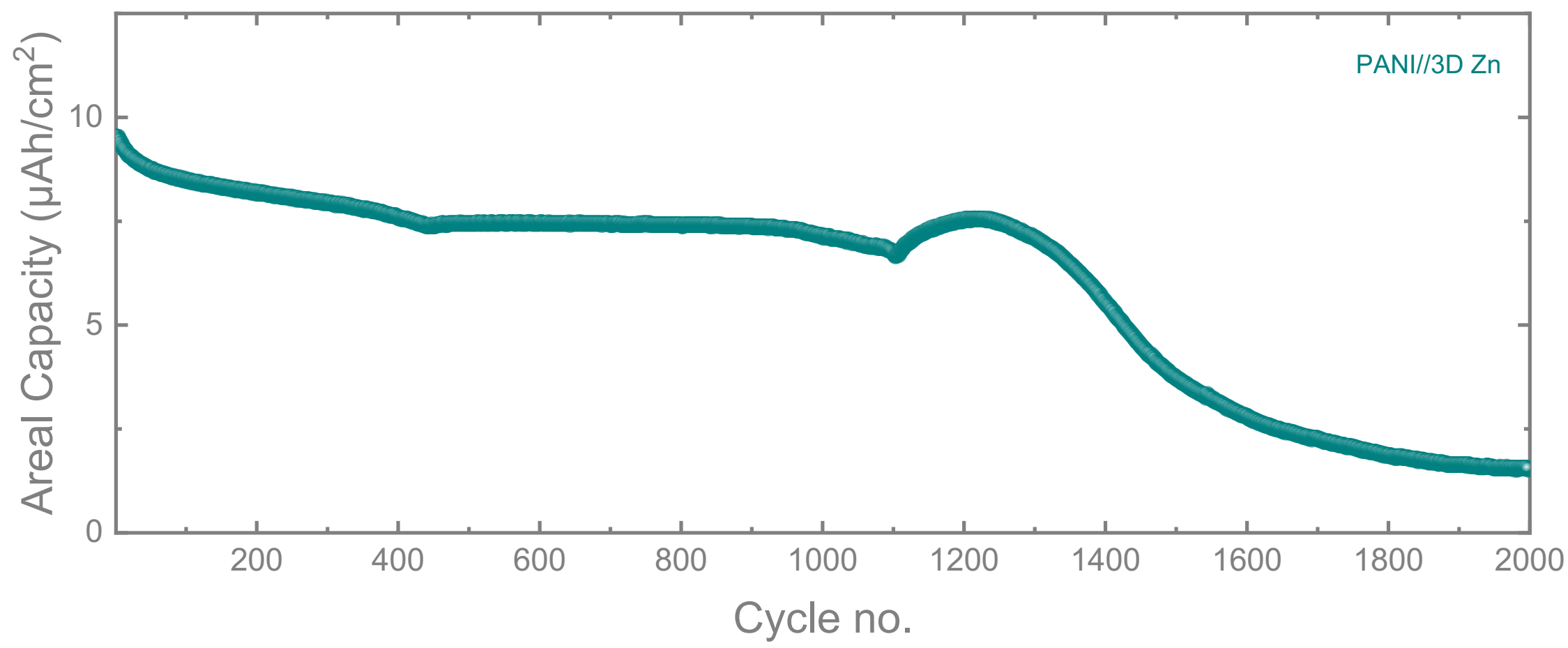

Figure S12. *Long cycling curve of PANI//3D Zn MBs in 2000 cycles.*

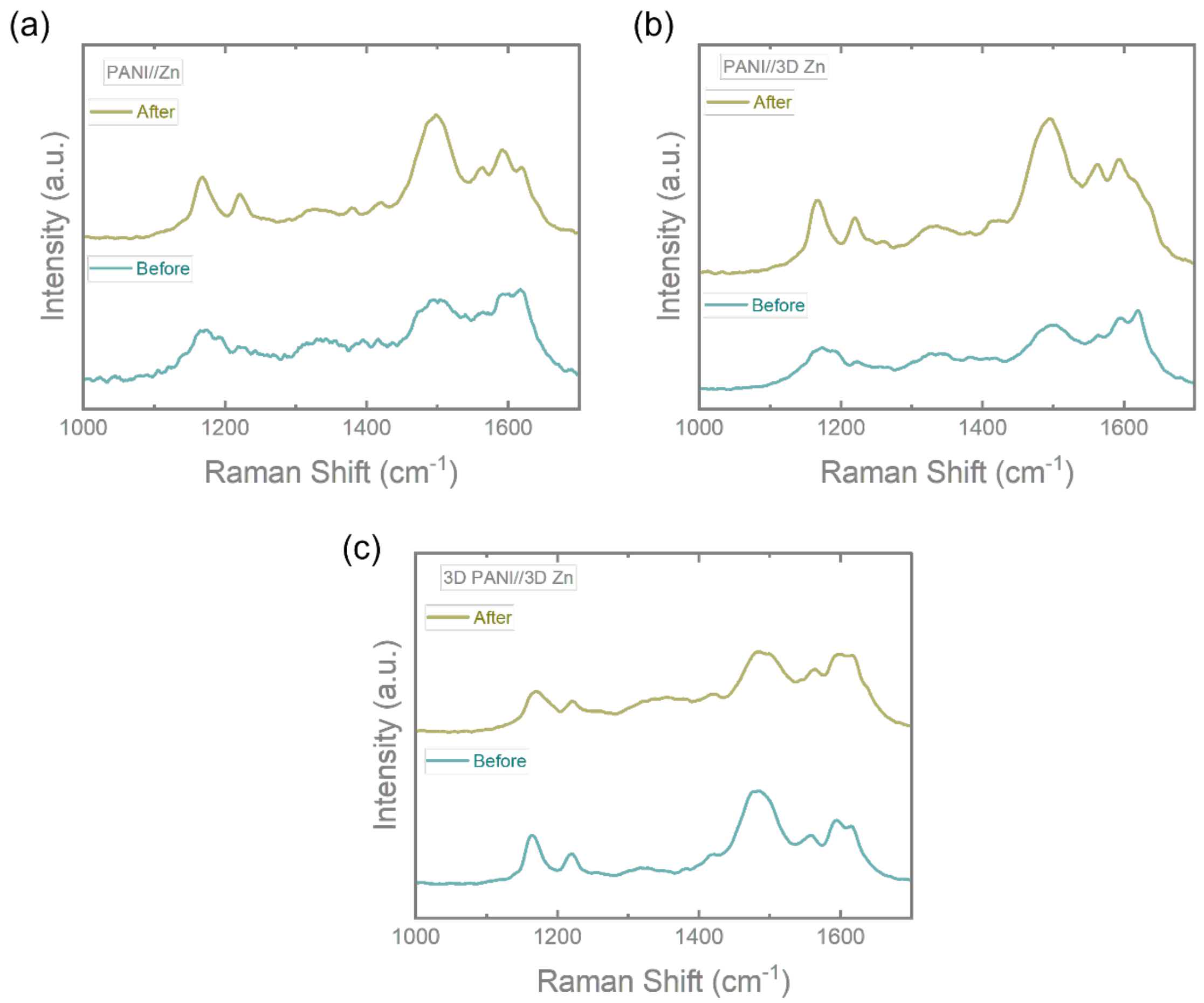

Figure S13. *Raman spectra of cathode sides of (a) ZIMB, (b) PANI//Zn(3D), and (c) 3D ZIMB before and after 2000 cycles.*

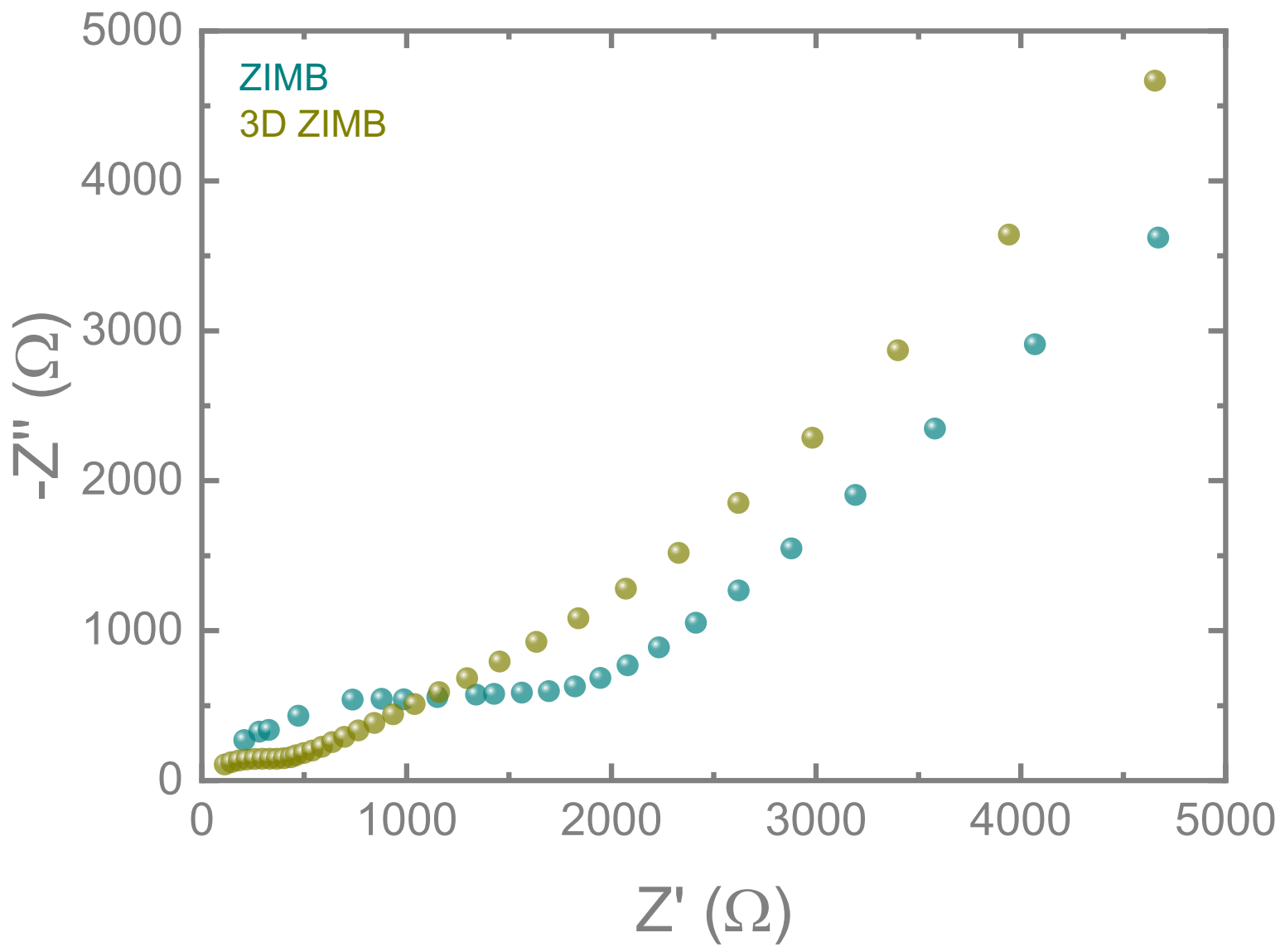

Figure S14. *EIS curves of ZIMB and 3D ZIMB after 2000 cycles.*

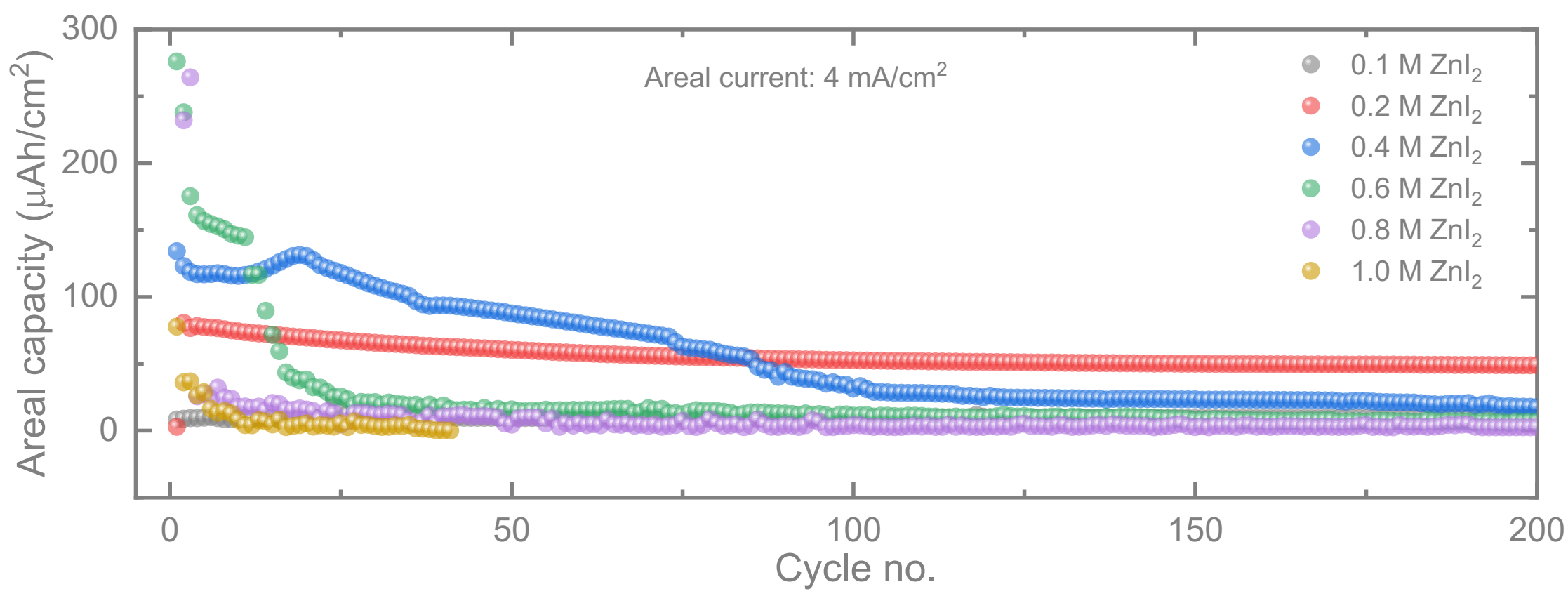

Figure S15. *Cycling performance of Zn//I_2 MBs in electrolyte with different ZnI_2 concentration.*

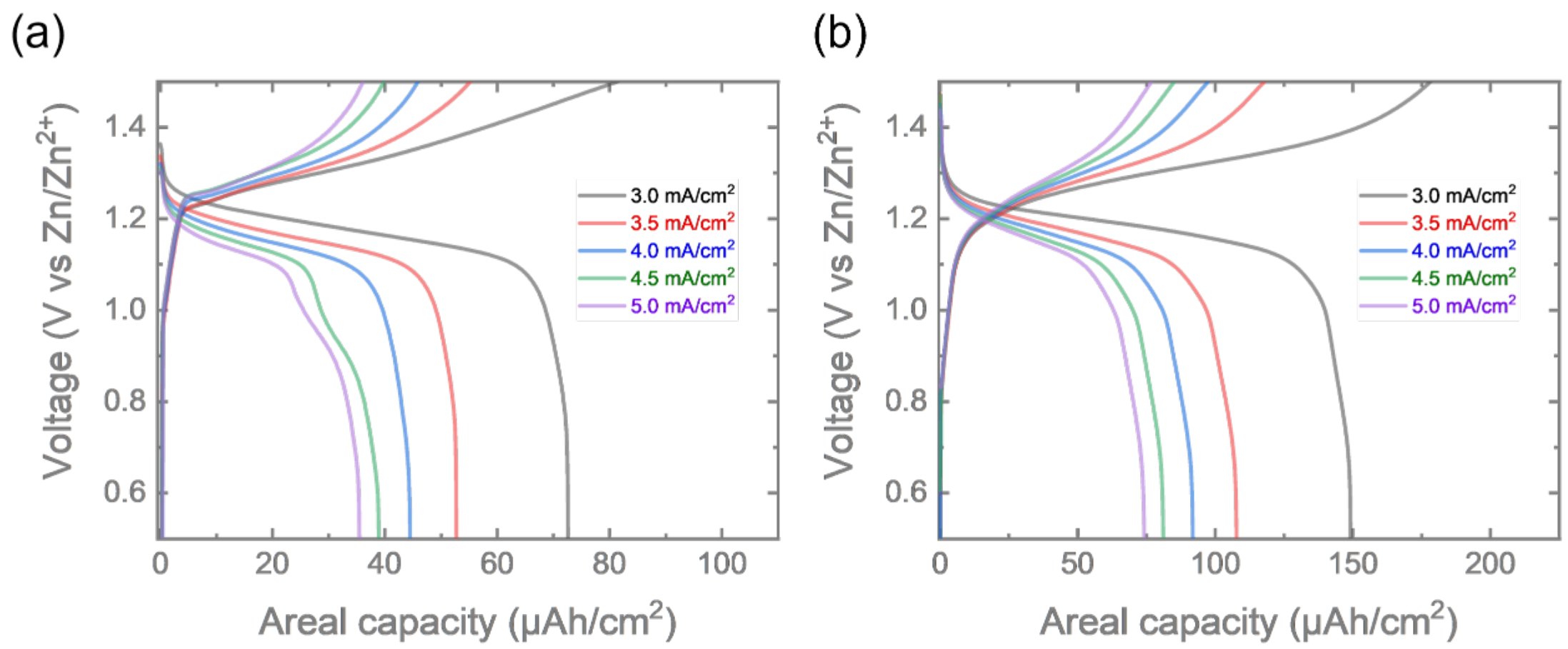

Figure S16. *GCD curves of (a) Zn//I_2 and (b) 3D Zn//I_2 MBs at different current densities.*

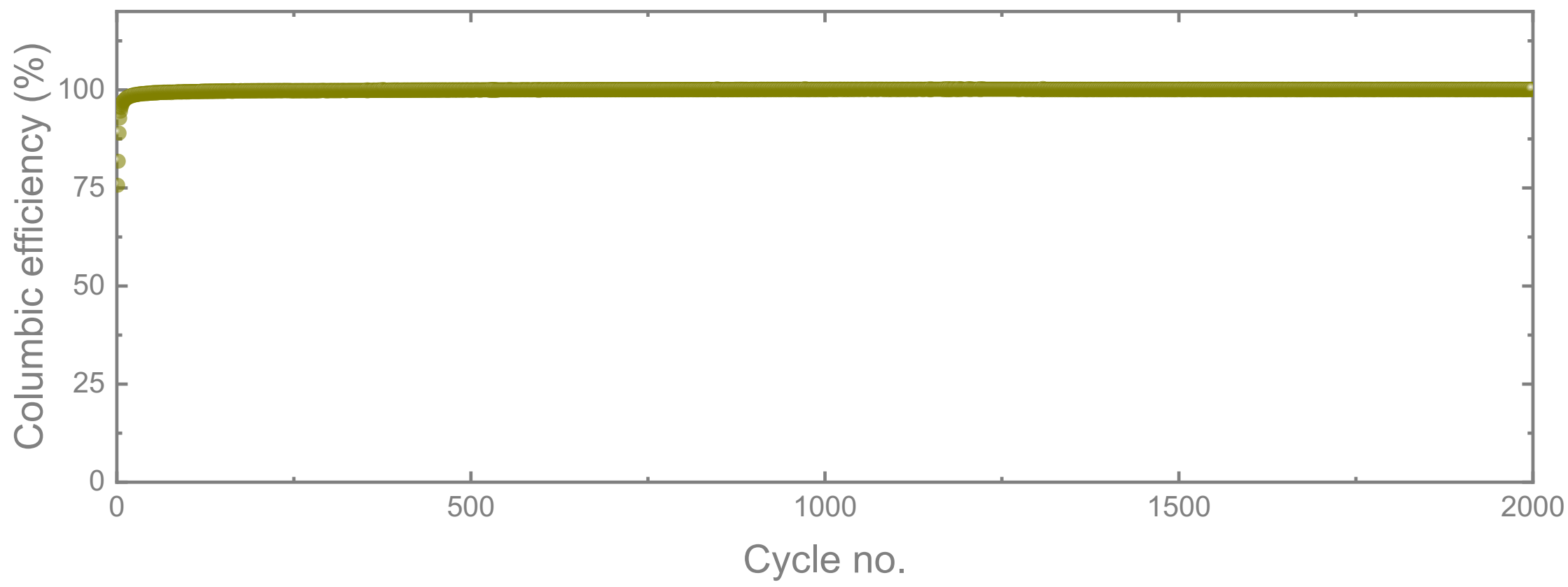

Figure S17. *Columbic efficiency of 3D Zn//I_2 MB during long cycling.*

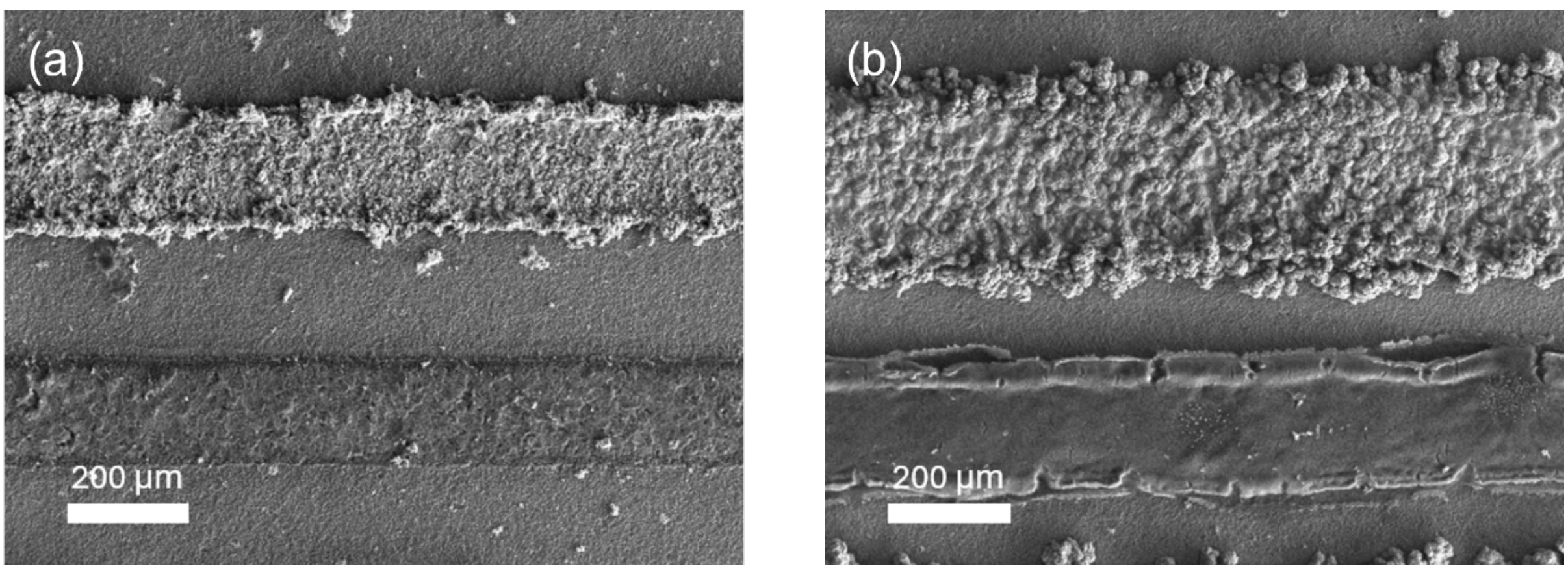

Figure S18. *SEM images of (a) Zn//I_2 and (b) 3D Zn//I_2 MBs after 250 cycles.*

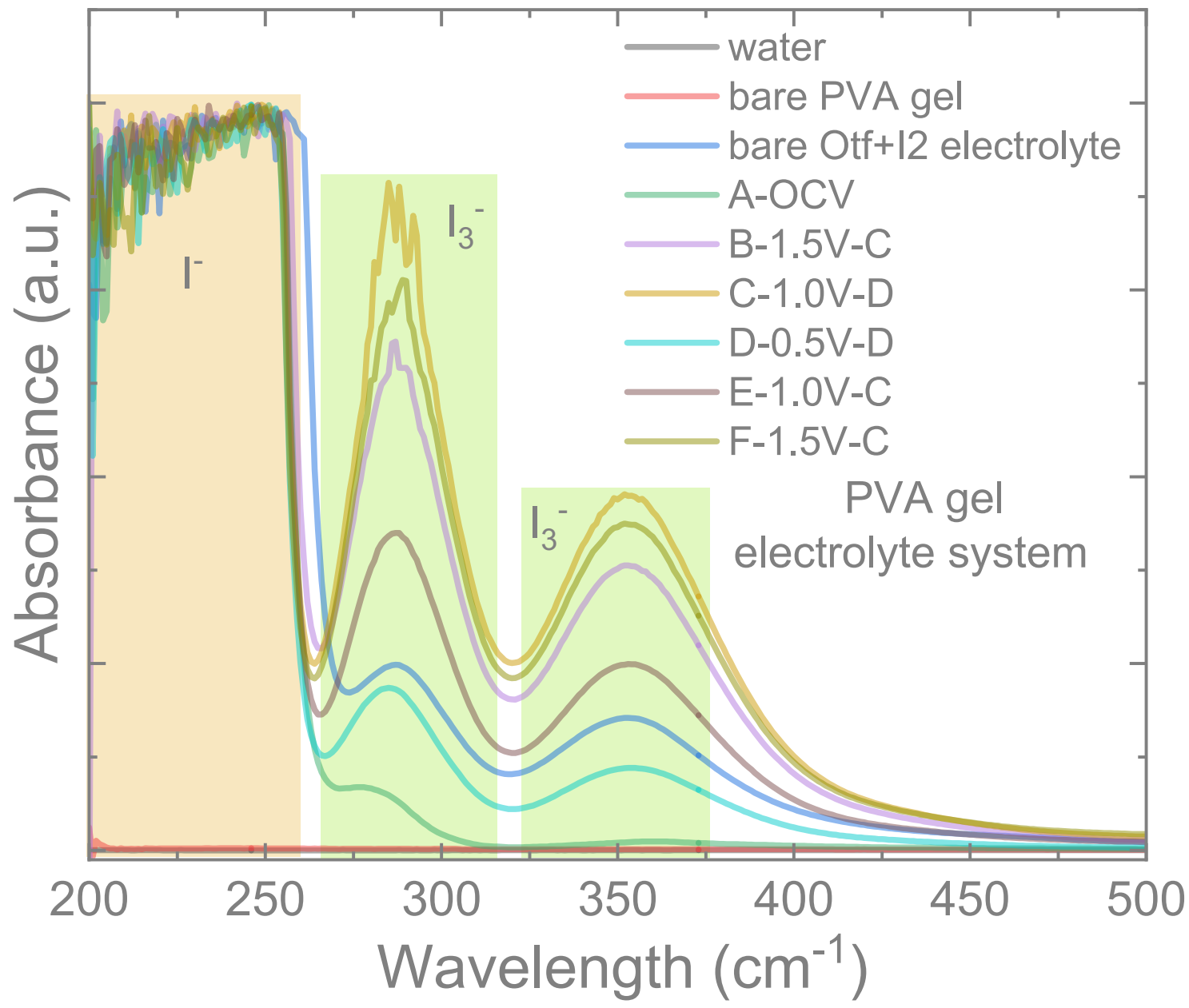

Figure S19. *Ex-situ UV–vis spectra of the PVA gel electrolyte collected at different states of charge for Zn//I_2 MBs: pristine PVA gel electrolyte, and PVA gel electrolyte at points A (OCV), B (1.5 V), C (1.0 V), D (0.5 V), E (1.0 V), and F (1.5 V), corresponding to the SoC process shown in* ***Figure 6a****.*

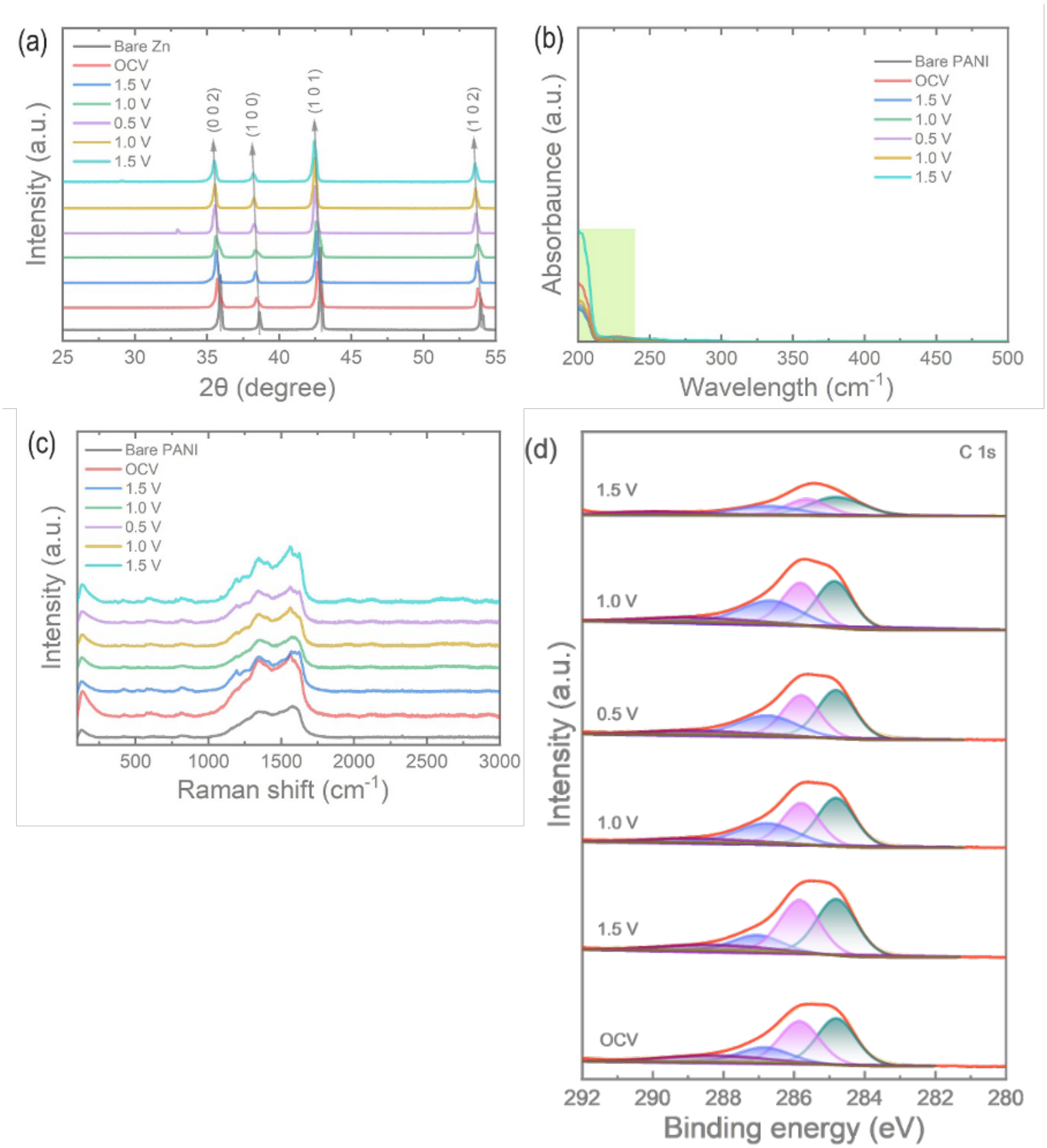

Figure S20. *(a) Ex-situ XRD curves for bare Zn, Zn electrodes at OCV, 1.5 V, 1.0 V, 0.5 V, 1.0 V, and 1.5 V in ZIMBs. (b) Ex-situ UV-vis for bare PANI, PANI electrodes at OCV, 1.5 V, 1.0 V, 0.5 V, 1.0 V, and 1.5 V in ZIMBs. (c) Ex-situ Raman spectra of bare PANI, PANI electrodes at OCV, 1.5 V, 1.0 V, 0.5 V, 1.0 V, and 1.5 V in ZIMBs. (d) Ex-situ XPS spectra of C 1s of the electrodes in Zn//I_2 MBs.*

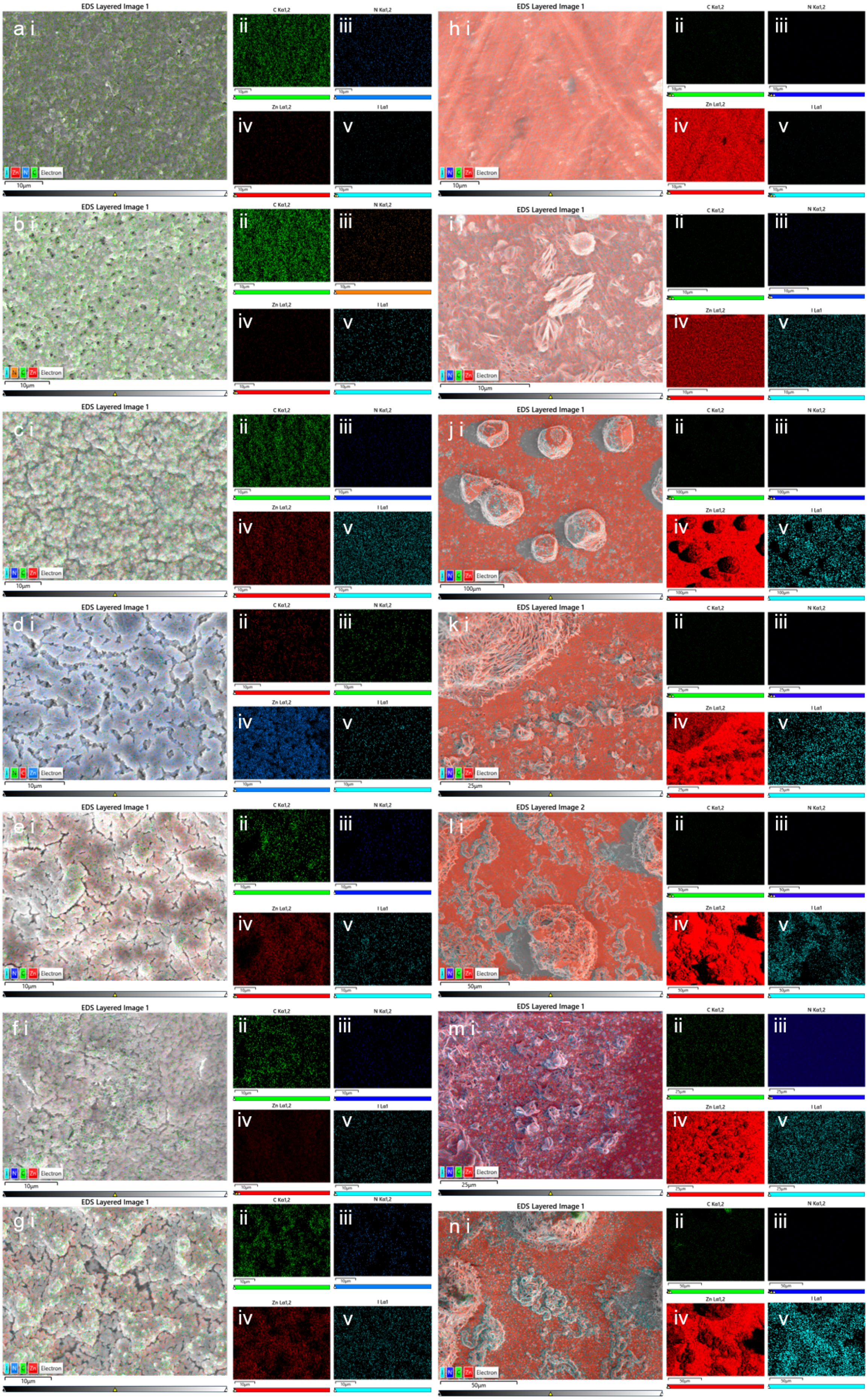

EDS Layered Image 1
EDS Layered Image 2
C Kα1,2
N Kα1,2
Zn Lα1,2
I Lα1
a i
b i
c i
d i
e i
f i
g i
h i
i i
j i
k i
l i
m i
n i
ii
iii
iv
v
Electron
10μm
25μm
50μm
100μm

Figure S21. *(i) Ex-situ SEM and EDS results of (ii) C, (iii) N, (iv) Zn, and (v) I for (a-g) cathodes and (h-n) anodes in Zn//*I_2 *MBs at different SoC of A-F.*
